# Dynamic DNA Assemblies in Biomedical Applications

**DOI:** 10.1002/advs.202000557

**Published:** 2020-06-08

**Authors:** Yaqin Hu, Ying Wang, Jianhua Yan, Nachuan Wen, Hongjie Xiong, Shundong Cai, Qunye He, Dongming Peng, Zhenbao Liu, Yanfei Liu

**Affiliations:** ^1^ Department of Pharmaceutical Engineering College of Chemistry and Chemical Engineering Central South University Changsha Hunan 410083 P. R. China; ^2^ Xiangya School of Pharmaceutical Sciences Central South University Changsha Hunan 410013 P. R. China; ^3^ Department of Medicinal Chemistry School of Pharmacy Hunan University of Chinese Medicine Changsha Hunan 410013 P. R. China; ^4^ Molecular Imaging Research Center of Central South University Changsha Hunan 410013 P. R. China

**Keywords:** biomedical applications, DNA nanotechnology, dynamic DNA assemblies

## Abstract

Deoxyribonucleic acid (DNA) has been widely used to construct homogeneous structures with increasing complexity for biological and biomedical applications due to their powerful functionalities. Especially, dynamic DNA assemblies (DDAs) have demonstrated the ability to simulate molecular motions and fluctuations in bionic systems. DDAs, including DNA robots, DNA probes, DNA nanochannels, DNA templates, etc., can perform structural transformations or predictable behaviors in response to corresponding stimuli and show potential in the fields of single molecule sensing, drug delivery, molecular assembly, etc. A wave of exploration of the principles in designing and usage of DDAs has occurred, however, knowledge on these concepts is still limited. Although some previous reviews have been reported, systematic and detailed reviews are rare. To achieve a better understanding of the mechanisms in DDAs, herein, the recent progress on the fundamental principles regarding DDAs and their applications are summarized. The relative assembly principles and computer‐aided software for their designing are introduced. The advantages and disadvantages of each software are discussed. The motional mechanisms of the DDAs are classified into exogenous and endogenous stimuli‐triggered responses. The special dynamic behaviors of DDAs in biomedical applications are also summarized. Moreover, the current challenges and future directions of DDAs are proposed.

## Graphical abstract

Recently, dynamic deoxyribonucleic acid (DNA) assemblies have emerged as promising platforms for diagnostics and therapeutics in biomedical applications. This work provides an overview of dynamic DNA assemblies, detailing their designing software, motional mechanisms, and dynamic behaviors in biomedical applications. The mechanisms are classified into exogenous and endogenous stimuli‐triggered responses. This work also summarizes this area of research and proposes future directions.

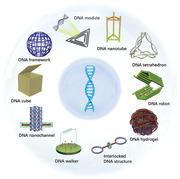

## 1 Introduction

Over the past decades, deoxyribonucleic acid (DNA) has shown usefulness as a specific material for constructing nanoscale machines.^[^ ^1^ ^]^ Through specific designing, DNA structures can be constructed due to the predictable assembly ability of DNA via Watson–Crick base pairing. In addition, DNA is easy to be synthesized and modified with other moieties.^[^ ^2^ ^]^ Some specific designed DNA assemblies showed the ability to simulate molecular motions and fluctuations in bionic systems. Owing to these unique properties, DNA nanotechnology and DNA assemblies are becoming increasingly important in the biomedical filed.

DNA nanotechnology was first elaborated by Seeman in the early 1980s.^[^ ^3^ ^]^ In 1982, they proposed Holliday junction, which was a mobile branched nucleic acid structure containing four double‐stranded arms joined together. After that, various DNA structural units were created,^[^ ^4^ ^]^ and sundry DNA nanostructures were emerged in succession.^[^ ^5^, ^6^, ^7^ ^]^ In 2004, a nanoscale octahedron was generated by folding a 1669‐nucleotide single‐stranded DNA (ssDNA) in the presence of five 40‐mer oligodeoxynucleotides through a simple denaturation–renaturation procedure,^[^ ^8^ ^]^ which laid the foundation of epoch‐making DNA origami technique, also called preorigami technique. In 2006, Rothemund presented a versatile and simple “one pot” method which used numerous staple strands to guide the folding of a 7 kb single‐stranded scaffold into various shapes such as square, rectangle, star, smile face, even map of western hemisphere, etc. These structures are roughly 100 nm in diameter and have a spatial resolution of about 6 nm.^[^ ^9^ ^]^ DNA origami technique is a milestone in the history of DNA nanotechnology, which enables the construction of DNA assemblies with more sophisticated and rigid structures. Nanoscale DNA structures, including analogic China map,^[^ ^10^ ^]^ dolphin‐like structure,^[^ ^11^ ^]^ DNA tweezer,^[^ ^12^ ^]^ DNA box,^[^ ^13^ ^]^ DNA polyhedron,^[^ ^14^ ^]^ etc., were created. Precisely tailored size and structural diversity enabled DNA origami as templates for forming crystal superlattices and constructing inorganic nanostructures.^[^ ^15^ ^]^ Besides, the salient addressability of DNA origami allows precise control over the location of the cargo, which can be used for optical super‐resolution imaging^[^ ^16^, ^17^ ^]^ and accurate arrangement of cargos.^[^ ^18^, ^19^ ^]^

In this review, we focus on working mechanisms and biomedical applications of dynamic DNA assemblies (DDAs). DDAs are DNA nanostructures that can perform structural transformations or predictable behaviors in response to corresponding stimuli. These features can be applied to control chemical reactions,^[^ ^18^ ^]^ construct reconfigurable plasmonic systems,^[^ ^20^, ^21^ ^]^ serve as drug delivery systems (DDSs),^[^ ^22^, ^23^ ^]^ etc. Herein, we divided DNA nanostructures into different types based on structural characteristics, and introduced their assembly principles. The related computer‐aided software was also summarized. Then, we elaborated on the switchable mechanisms of DDAs and relevant applications. In addition, according to the special behavioral features and application characteristics, we specifically introduced DNA walker, DNA nanochannel, DNA hydrogel, DNA points accumulation in nanoscale topography (DNA‐PAINT), etc. The current challenges and future directions were also discussed.

## 2 Assembly of DNA Nanostructures

**Figure 1:**
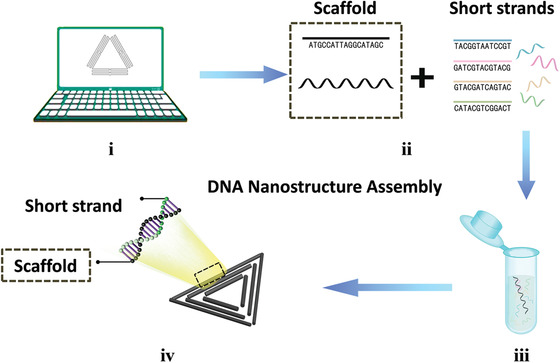
DNA nanostructure assembly. i) Designing DNA nanostructure with computer‐aided software to determine the sequences of all strands. ii) Preparation of all materials. iii) Self‐folding of strands through annealing. iv) Generation of targeted DNA nanostructures.

The “one pot” method is the basic approach for DNA nanostructure assembly (**Figure** **1**).^[^ ^9^ ^]^ The first step is to design DNA nanostructure model with special auxiliary software to ascertain the sequences of all DNA strands. After that, all strands are synthesized and mixed in specific buffer to fold into designed shapes under rigorous annealing. Additionally, the assembly of DNA origami structures contains the usage of DNA scaffold.

### 2.1 Closely Packed DNA Origami Nanostructures (CPDONs)

**Figure 2:**
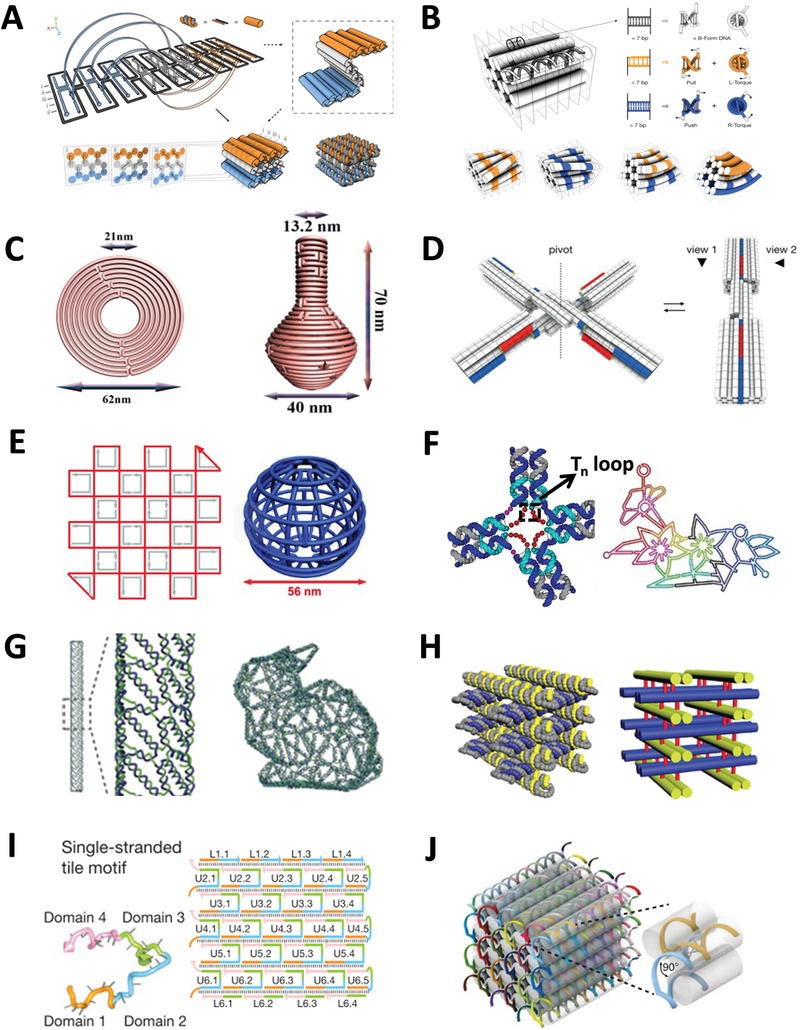
CPDONs, WDONs, and DNADUs. A) 3D DNA origami based on honeycomb lattice. Reproduced with permission.^[^ ^24^ ^]^ Copyright 2009, Nature Publishing Group. B) Design principles for controlling twist and curvature in DNA bundles. Reproduced with permission.^[^ ^26^ ^]^ Copyright 2009, American Association for the Advancement of Science. C) DNA origami nanostructures with complex curvatures: a nine‐layer concentric ring structure and a nanoflask. Reproduced with permission.^[^ ^27^ ^]^ Copyright 2011, American Association for the Advancement of Science. D) Reversible reconfiguration of shape‐complementary DNA objects. Reproduced with permission.^[^ ^29^ ^]^ Copyright 2015, American Association for the Advancement of Science. E) DNA gridiron nanostructures based on four‐arm junctions. Reproduced with permission.^[^ ^14^ ^]^ Copyright 2013, American Association for the Advancement of Science. F) WDONs with multiarm junction vertices: *T*_n_ loop in DNA nanostructure and a flower‐and‐bird pattern. Reproduced with permission.^[^ ^34^ ^]^ Copyright 2015, Nature Publishing Group. G) 3D meshes rendered in DNA: a rod and a version of the Stanford bunny. Reproduced with permission.^[^ ^35^ ^]^ Copyright 2015, Nature Publishing Group. H) 3D framework DNA origami nanostructures with layered crossovers. Reproduced with permission.^[^ ^36^ ^]^ Copyright 2016, Wiley‐VCH Verlag. I) ssDNA tile strategy. Reproduced with permission.^[^ ^38^ ^]^ Copyright 2012, Nature Publishing Group. J) DNA module strategy. Reproduced with permission.^[^ ^39^ ^]^ Copyright 2012, American Association for the Advancement of Science.

The feature of CPDONs is close‐packed helices. Three breakthroughs are involved in the development of DNA origami technique. The first is the transition from 2D to 3D structure. In 2009, Douglas et al. extended the “one pot” method to build 3D shapes formed by layers of DNA helices that arranged into honeycomb lattices (**Figure** **2**A).^[^ ^24^ ^]^ The linkages between adjacent DNA helices formed crossovers to guide right folding. Based on that principle, they designed six shapes including monolith, square nut, bridge, genie bottle, stacked cross, and slotted cross. The dimensions of these nanostructures were precisely controlled within the range from 10 to 100 nm. Soon after, Douglas's group described a more compact structure called square lattice,^[^ ^25^ ^]^ which was more flat than honeycomb lattice and more suitable to fabricate densely packed architectures. The honeycomb and square lattice are basic models for subsequent 3D CPDONs.

The second is the development from rigid geometry to twisted and curved shapes. Dietz et al. controlled the twist and curvature of DNA helix by positionally inserting or deleting base pair in the bundle, demonstrating that the feasibility of the construction of CPDONs with twist and curvature (Figure 2B).^[^ ^26^ ^]^ Later, Han et al. proposed a strategy for constructing CPDONs with more intricate curved surfaces by adding an array of crossovers between adjacent helices. They successfully verified the strategy on 2D shapes like concentric ring and concentric square frame structure, and complex 3D DNA architectures like hemisphere, sphere, ellipsoidal shell, etc. (Figure 2C).^[^ ^27^ ^]^ The verification of the concept of curved surface made DNA origami technique a big step forward and endowed DNA nanostructures with new functionalities, such as mimicking structural and functional features of proteins.^[^ ^28^ ^]^

The third is the emergence of shape‐complementarity theory. The fundamental principle of DNA assembly is usually complementary base pairing. However, in 2015, Gerling et al. added a new chapter to the assembly of DNA origami.^[^ ^29^ ^]^ They demonstrated that discrete 3D DNA components with complementary concave/convex domains could achieve specific self‐assembling based on shape‐complementarity without DNA base pairing (Figure 2D), which was similar to stacking toy bricks. These multidomain assemblies were stabilized via short‐ranged nucleobase stacking bonds that compete against electrostatic repulsion between the components’ interfaces. The conformation of DNA assemblies is unstable due to the weak base stacking interaction. To achieve shape‐complementarity of DNA assemblies, salt concentration of buffer is critical. This breakthrough enabled the construction of structural switchable DNA nanostructures and large DNA assemblies.

Due to tightly packed DNA helices, CPDONs are stable to serve as drug carriers.^[^ ^30^ ^]^ The shape plasticity makes CPDONs hold great potential in the construction of other materials, such as the templates for creating plasmonic structures and nanophotonic devices.^[^ ^31^ ^]^ Additionally, CPDONs with salient addressability provide excellent platforms for accurate arrangement of cargos.^[^ ^32^, ^33^ ^]^

### 2.2 Wireframe DNA Origami Nanostructures (WDONs)

Before the emergence of DNA origami technique, wireframe DNA nanostructures were already existed, of which, the structures are usually simple, such as tetrahedron.^[^ ^7^ ^]^ In 2013, Han et al. first created 2D and 3D wireframe DNA nanostructures as the basic structural units by DNA origami technique, in which, a set of four‐arm junctions analogous was used to stabilize the Holliday junctions (Figure 2E).^[^ ^14^ ^]^ However, WDONs assembled in this way are symmetrical, lacking of diversity and flexibility. Later, a more flexible strategy for fabricating WDONs with multiarm junctions was proposed by Zhang et al. (Figure 2F).^[^ ^34^ ^]^ In fact, the design principle of both methods is connecting all multiarm junctions together to form the scaffold pathway first, and then fixing the scaffold by designed staple strands sequences, finally adjusting the angles between arms to reduce structural tension. The cleverness of Zhang's strategy is that the angles between adjacent edges can be adjusted by *T*_n_ loop with appropriate length, which makes WDONs get rid of the limitation of symmetrical shapes. WDONs based on Zhang's strategy were more stable because the edges were double helixes. In 2015, the technology for fabricating WDONs was further expanded by Benson et al.^[^ ^35^ ^]^ They presented a general method based on A‐trails routing theory (a specific type of Eulerian circuits) to construct large WDONs with minimal amount of DNA. A‐trail algorithm determines the route of scaffold, which means that the scaffold strand traversed every edge of structure along nonknotted paths and all vertices of WDONs have a uniform degree (Figure 2G). A series of successfully prepared WDONs, such as hollow ball, nicked torus, bottle, and the Stanford bunny had verified the feasibility of this method. Then, Hong et al. reported a novel strategy to engineer multilayered wireframe DNA structures by introducing layered crossovers to joint neighboring layers, which further enriched WDONs (Figure 2H).^[^ ^36^ ^]^

Traditionally, CPDONs are easier to be observed than WDONs when imaged using atomic force microscopy (AFM) or transmission electron microscopy (TEM). However, WDONs can be easily incorporated with DNA molecules into addressable edges. Additionally, hollow DNA nanostructure is preferential for biological applications, which have the structural flexibility and large space for payload and closed protective structures. WDONs with unique curved shape can also be used to imitate the morphology of biomolecules.^[^ ^37^ ^]^

### 2.3 DNA Nanostructures Assemblied by DNA Units (DNADUs)

Large DNA supramolecular architectures are difficult to construct because scaffold with long length is easy to break, not to mention the workload of a plenty of staple strands. Therefore, the construction of large DNA nanostructures generally adopts the combination of DNA components. ssDNA tile^[^ ^38^ ^]^ and DNA module^[^ ^39^ ^]^ with increased flexibility have been implemented into the construction of nanostructures (Figure 2I,J). The main feature of the combined strategy is that large arbitrary DNA shapes could be constructed quickly by modularized DNA units without long scaffold strand. For example, by adjusting the stiffness and geometry of build‐blocks, the shape and mechanical properties of DNA nanotube will change.^[^ ^40^ ^]^ Small DNA units are more convenient and flexible than the long scaffold because they can be removed and added independently. However, the yields and stabilities of DNA nanostructures were significantly reduced. Mohammed and Schulman invented a seed growth method, where a DNA origami tube was employed as a seed to guide the growing of DNA tiles to form DNA nanotube with controllable size.^[^ ^41^ ^]^ A DNA nunchuck was constructed by seed growth method.^[^ ^42^ ^]^ The nunchuck consists of two nanotubes that grow from two growth sites on a nunchuck seed. Seed growth could be potentially used for spatially and temporally control of nanotube nucleation, and be extended to form more complex arrangements of nanotubes or other DNA nanostructures.

Linkers between DNA units are easily to be broken or disintegrated by the influence of salt or temperature, which lead to the configuration change of DNADUs. Therefore, by observing configuration conversion, DNADUs can be applied to detect the changes of environmental salt concentration and temperature.^[^ ^43^ ^]^ However, DNADNs are seldom applied in vivo due to their poor stability.

### 2.4 Interlocked DNA Nanostructures (IDNs)

**Figure 3:**
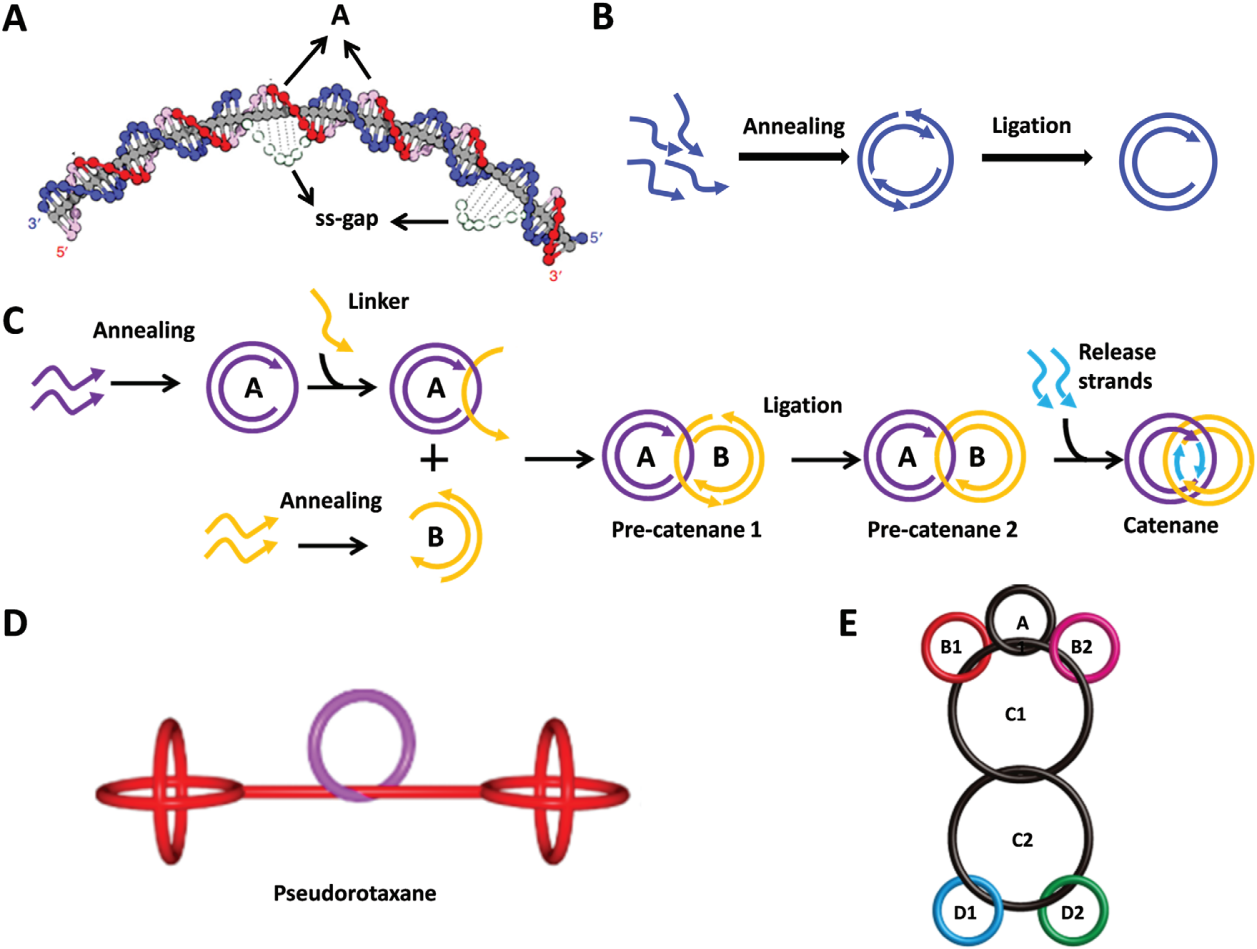
IDNs. A) The structural characteristics and formation mechanisms of dsDNA ring, B) The assembly of DNA ring, and C) The assembly of DNA catenane. Reproduced with permission.^[^ ^47^ ^]^ Copyright 2019, Nature Publishing Group. D) A pseudorotaxane nanostructure. Reproduced with permission.^[^ ^48^ ^]^ Copyright 2012, American Chemical Society. E) A molecular robot consisting of seven rings. Reproduced with permission.^[^ ^49^ ^]^ Copyright 2015, American Chemical Society.

IDNs are composed of two or more components that are linked to each other by linkage rather than by chemical bonds. Although the components cannot dissociate, relative movement between components confers the structure with great flexibility. IDNs were initially assembled from ssDNA,^[^ ^44^ ^]^ and developed into dsDNA interlocked architectures with better shape persistence.^[^ ^45^ ^]^ The substructures of IDNs contain rings, spherical stoppers, and axles, in which the curvature of rings is induced by repetitive, intrinsically bent poly A‐tracts (**Figure** **3**A). The assembly of ring is different from the general nanostructures. The ring assembled by staple strands is not stable enough. Therefore, ligation step is conducted at 15 °C following annealing to ligate staple strands to stabilize the structure. Specifically, the 5′ ends of the strands used for ligation are phosphate‐modified for forming relatively long strand with the aid of ligase (Figure 3B).

Obviously, it is impossible to synthesize interlocked nanostructure by “one pot” method due to the multiple components. The synthesis must be performed in a strict order, which requires that the melting temperature of the whole structure is higher than annealing temperatures. The ss‐gap regions of the interlocked assembly are essential to substructures, which are the junction sites. The specific assembly process is illustrated in Figure 3C, where catenane was employed as an example. Both prering A and B were assemblied in “one‐pot” reaction, and then pre‐ring B was threaded into pre‐ring A with the linker strand and ligated to form pre‐catenane 1. ss‐gap was the complementary sequence between linker and pre‐ring A. Pre‐catenane 2 was generated after ligation. The release strands are added to release pre‐ring A and B to form catenane, and intact rings A and B were formed. Catenanes, rotaxane, and daisy‐chain rotaxane are classic IDNs.^[^ ^46^, ^47^ ^]^ Interlocked structures assembled through slippage process, the temperature, ring size, length, and mismatch position have a significant impact on the efficiency of formation (Figure 3D).^[^ ^48^ ^]^

Compared to CPDONs, WDONs, and DNADUs, IDNs with greater flexibility are promising for constructing DDAs with increasingly complex and higher controllable functions. The motion of interlocked elements can be controlled by noncovalent interactions. IDNs can act as DNA molecular motors and switches to perform specific tasks under chemical or physical stimuli. For instance, a seven‐ring interlocked DNA catenane system displayed the unique ability of reversible switchable reconstruction in the presence of fuel strands and antifuel strands (Figure 3E).^[^ ^49^ ^]^

## 3 Computer‐Aided Software for Designing DNA Nanostructures

Designing DNA nanostructures is quite complicated and cumbersome. Staple strands screening and thousands of base pairs and heavy calculations made the design a vast project. Therefore, computer‐aided software was invented to reduce workload. Here, some commonly used software is summarized according to their applicable features.

### 3.1 Software for General DNA Nanostructure Designing

#### 3.1.1 Nucleic Acid Package (NUPACK)

NUPACK is a basic software that specializes in the analysis and design of nucleic acid secondary structures of systems containing one or more interacting strands.^[^ ^50^ ^]^ It has three functions: analysis, design, and utilities. As long as the sequences of DNA strands are inputted, the partition function and minimum free energy (MFE) of secondary structure of all possible complexes of nucleic acid will be calculated. Concentrations of interacting strands and assembly temperature are also considered. However, the number of strands that NUPACK can simultaneously analyze is limited.

#### 3.1.2 Graphical Integrated Development Environment for Oligonucleotides (GIDEON)

GIDEON is a relatively systematic rendering program for designing DNA nanostructures.^[^ ^51^ ^]^ It allows directly model construction and provides 3D view. Also, GIDEON has the ability to predict the actual distortion of DNA nanostructures. However, there is an obvious shortage of GIDEON, in which, the sequence generation is not integrated with the modeler, resulting in the DNA sequences need to be determined by another auxiliary program. Additionally, the structure data of GIDEON were not rich enough to support large and complex designs.

#### 3.1.3 Tiamat and NanoEngineer‐1

Tiamat makes up for two major flaws in GIDEON, which is equipped with the sequence generation, rich data structures and flexible visualization strategies.^[^ ^52^ ^]^ NanoEngineer‐1 is a multifunctional molecular modeling and simulation system. Both two programs are powerful 3D editing tools for modeling large and accurate composite systems, in which, NanoEngineer‐1 can be used to construct carbon nanotubes model, mechanical components, and other molecular machines as well.

### 3.2 Software for CPDONs Designing

#### 3.2.1 Semiautomated Scientific Data Editor (SARSE)

The first computer‐aided software in origami design was SARSE developed by Andersen's team.^[^ ^11^ ^]^ A dolphin‐like DNA origami structure was designed with it. Atomic model can be inspected in a 3D viewer, and the 2D size of the shape is displayed to clearly reflect the precise position of each element of the structure. But the degree of automation of SARSE is not high enough.

#### 3.2.2 CaDNAno

CaDNAno, a powerful open‐source software specially designed for DNA origami based on honeycomb framework, is currently the most commonly used software for DNA origami design.^[^ ^53^ ^]^ Three interrelated panels of caDNAno provide detailed information of origami structure during the design process. Slice panel provides a cross‐sectional view of origami model. Path panel is used to edit the path of scaffold and staple strands, and displays DNA origami as an unrolled 2D schematic. Render panel shows a real‐time 3D model of origami structure. The scaffold folding path is determined according to the arrangement of double helix.

#### 3.2.3 CanDo

Differing from other design software, canDo is a computational tool based on caDNAno for studying complex objects containing curved and distorted elements.^[^ ^54^ ^]^ It generates an initial configuration by reading geometric data including helix location, locations of inserted and/or deleted base pairs, and crossover position. CanDo deforms adjacent helices to simulate a deformed configuration that shows as a heatmap indicating local root‐mean‐square‐fluctuations (RMSFs), based on which, the structural freedom and local flexibility of the DNA origami nanostructure can be predicted. CanDo predicts the rotation and reproduces the overall degree of torsional deformation within a 15% error.

### 3.3 Software for WDON Designing

#### 3.3.1 vHelix

The design concept of vHelix is dramatically different from software for CPDONs.^[^ ^35^, ^55^ ^]^ The polygon mesh is manually drawn in vHelix, and the scaffold path through all the edges of the wireframe is determined automatically by vHelix adapting a nonknotted path. Before converting into scaffold and staple sequences, there is a physical model for adjusting the distribution of strain to ensure the stability of the structure. However, the software's algorithm is not accurate, resulting in compatibility or instability of designed components, thus unable to design complex origami structures.

#### 3.3.2 DNA Origami Sequence Design Algorithm for User‐Defined Structures (DAEDALUS)

DAEDALUS designed by Veneziano et al. is a fully autonomous design procedure that programs arbitrary WDONs based on an input wireframe mesh without reliance on users’ feedback, and there is no limitation to spherical topologies.^[^ ^56^ ^]^ The algorithm of DAEDALUS is more stable than vHelix, and the application range is broader, however, the edges of the structures designed by vHelix and DAEDALUS are limited to DNA duplexes.

#### 3.3.3 Programmed Eulerian Routing for DNA Designs Using X‐Overs (PERDIX) and 3D, Algorithmically Generated Library of DNA Origami Shapes (TALOS)

PERDIX^[^ ^57^ ^]^ and TALOS^[^ ^58^ ^]^ for designing programming arbitrary 2D wireframe geometries with double‐stranded‐based edges and 3D wireframe geometries with 6‐helix bundle (6HB) per edge were invented by Jun et al. The most salient characteristic of these two software is that users only need to input the boundary of the designed wireframe DNA object, and the internal structure is determined automatically. The scaffold routings and sequence assignment of complementary staple strands will be completed. Software is summarized in **Table** **1**.

**Table 1:** Computer‐aided software

DNA nanostructures	Software	Functionalities	Disadvantages	Operative difficulties	Websites	Refs.
General DNA nanostructures and IDNs	NUPACK	Analysis: thermodynamic analysis of nucleic acid interaction Design: nucleic acid secondary structures Utilities: evaluation of equilibrium properties of nucleic acid complex	3D DNA structure is not supported	Easy	http://www.nupack.org	^[^ ^50^ ^]^
	GIDEON	Straightforward construction and viewing of simple models	Without generation of DNA sequences	Easy	/	^[^ ^51^ ^]^
	Tiamat	Simulation of large and complex DNA structures	Model parameters need to be enriched	Medium	http://chemistry.asu.edu/faculty/hao yan.asp	^[^ ^52^ ^]^
	NanoEngineer‐1	Dynamic model building for multiple materials	The types of DNA structures are limited	Difficult	https://github.com/kanzure/nanoengineer	/
CPDONs	SARSE	Designing of 2D DNA origami structures	Low degree of automation	Medium	http://www.cdna.dk/origami	^[^ ^11^ ^]^
	CaDNAno	Designing of 3D DNA origami shapes based on honeycomb frameworks	Origami types are limited	Difficult	http://cadnano.org/	^[^ ^53^ ^]^
	CanDO	Designing origami objects with curved and twisted elements	Twisted elements are limited	Difficult	http://cando.dna‐origami.org/	^[^ ^54^ ^]^
WDONs	vHelix	Designing 2D/3D WDONs	Unstable assemblies exist	Difficult	http://www.vhelix.net	^[^ ^35^, ^55^ ^]^
	DAEDALUS	Designing arbitrary topological spherical framework DNA structures	The edge of structure is limited to duplexes	Difficult	http://daedalus‐dna‐origami.org	^[^ ^56^ ^]^
	PERDIX	Designing DX‐based 2D DNA wireframe polyhedra	/	Medium	http://perdix‐dna‐origami.org	^[^ ^57^ ^]^
	TALOS	Designing 6HB‐based 3D DNA wireframe polyhedra	/	Medium	http://talos‐dna‐origami.org	^[^ ^58^ ^]^

## 4 Mechanisms of Dynamic Behaviors of DDAs in Biomedical Applications

The dynamic DNA structures were divided into two categories, structural switchable DNA nanostructures and DNA motors. The dynamic behaviors of DDAs triggered by specific stimuli, such as ion concentration, pH, light, and biomarkers, make DDAs own great potential in sensing, transportation, imaging, drug delivery, etc. Here, we introduce the mechanism of dynamic behavior of DDAs and relevant applications from both exogenous and endogenous stimuli aspects.

### 4.1 Exogenous Stimuli

#### 4.1.1 Ions

**Figure 4:**
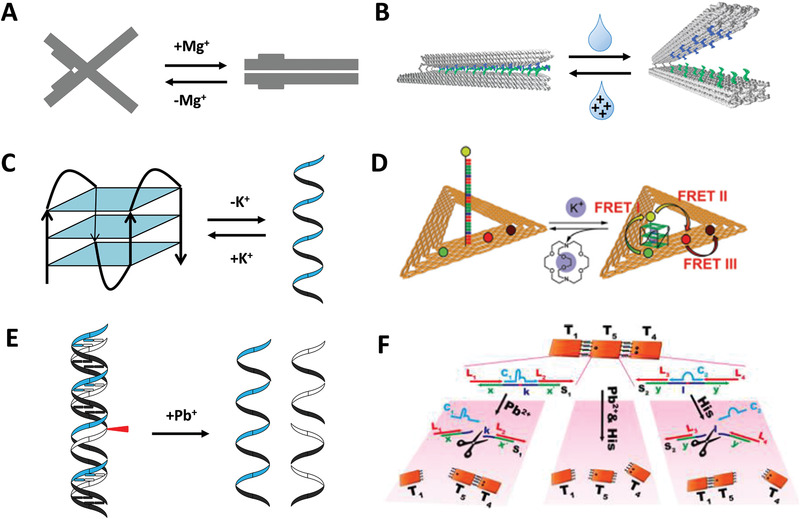
Ion actuation of DDAs. A) Salt actuation. B) A cation‐activated reconfigurable DNA origami. Reproduced with permission.^[^ ^61^ ^]^ Copyright 2018, American Chemical Society. C) Linear G‐rich telomeric DNA strands. D) A four‐color FRET photonic wire based on K^+^‐dependent G‐quadruplex. Reproduced with permission.^[^ ^65^ ^]^ Copyright 2016, The Royal Society of Chemistry. E) Pb^2+^ activated DNAzyme. F) DNA trimer system controlled by Pb^2+^ and histidine dependent DNAzymes. Reproduced with permission.^[^ ^69^ ^]^ Copyright 2016, American Chemical Society.

The response to ions is called ion actuation, which includes two types. The first is the electrostatic repulsion caused by the negative charges phosphates backbone under the condition of cations (**Figure** **4**A). Cations play significant roles during the assembly of DNA nanostructures particularly in the process of high‐density helical DNA origami, because intrinsic torsional stiffness of DNA, especially DNA supercoiling, is closely related to cation concentration. Additionally, proper cation concentration facilitates helix formation and tight arrangement of the helices.^[^ ^59^ ^]^ Reconfiguration of DNA assemblies of shape‐complementarity was controlled by changing the concentration of monovalent and divalent cations.^[^ ^29^ ^]^ The cation with different valences has different effects on DNA nanostructures, which depends on the distribution of ions, binding locations, and residence time across the structure.^[^ ^60^ ^]^ For instance, the adjusting ability of Na^+^ is much weaker than that of Mg^2+^ because Na^+^ is weakly bound to DNA origami and can be quickly exchanged in solvents. Thus, the ability of DNA backbone for negative charge screening is reduced, leading to the repulsion of parallel helices and instability of crossovers. Besides more effective binding ability, Mg^2+^ also features with long residence time at some specific locations, such as crossovers. Recently, Marras et al. constructed a cation‐activated reconfigurable DNA origami and studied the effect of mono‐, di‐, and trivalent ions on its behaviors (Figure 4B).^[^ ^61^ ^]^ Instead of shape‐complementarity principle, the reconfiguration was controlled by hybridization and dehybridization of mutually complementary short overhangs on DNA hinge inside. The hybridization of short overhangs was susceptible to ion concentration. The sensitivity of the device to the actuation increases dramatically with the increased valence of ions, and the ratio of the closed state is positively correlated with the ion concentration.

The second ion actuation relies on ion‐stimulated molecules. Linear G‐rich telomeric DNA strands that fold into G‐quadruplex structures in the presence of specific cations such as K^+^ or Na^+^, and return to single‐stranded structures when cations are removed (Figure 4C).^[^ ^62^, ^63^ ^]^ G‐quadruplex is formed by continuously stacked G‐quartets that are planar ring structures stabilized by Hoogsteen bonds among four guanines that can be provided by one, two, or four independent strands. The metal ion dependence is attributed to that the coordination of the O6 guanine atoms in G‐quartets and metal ions can stabilize the G‐quadruplex, which makes G‐quadruplex a powerful tool for ion detection.^[^ ^63^ ^]^ For example, Na^+^ or K^+^‐dependent G‐rich DNA sequences were used to be incorporated into DNA origami pliers, and the existence of Na^+^ or K^+^ was judged by the reconfiguration of the pliers.^[^ ^64^ ^]^ Olejko et al. applied K^+^‐selective G‐quadruplex in DNA origami triangle realizing a reversibly switchable three and four‐color fluorescence resonance energy transfer (FRET) cascade based on the distance effect of FRET (Figure 4D).^[^ ^65^ ^]^ DNAzyme, ssDNA with catalytic function, is able to catalyze many biochemical reactions, thus being widely used in asymmetric catalysis, biosensors, DNA nanotechnology, and diagnostics.^[^ ^66^ ^]^ There are ion‐dependent DNAzymes that cleave phosphodiester skeleton at nucleotide or deoxynucleotide sites in the presence of corresponding ions (Figure 4E). Up to date, DNAzymes special for Pb^2+^,^[^ ^66^ ^]^ Zn^2+^,^[^ ^67^ ^]^ Ag^+^,^[^ ^68^ ^]^ etc., have been developed. Wu et al. functionalized DNA origami tile with Pb^2+^ and histidine dependent DNAzyme to manipulate the dissociation of dimers and trimers by controlling the addition of Pb^2+^ and histidine (Figure 4F).^[^ ^69^ ^]^

Ion‐mediated DDAs exhibit different states by adjusting the salt concentration which realized rapid reconfiguration on millisecond time scales. Taking advantage of the feature, salt‐sensitive DNA devices can serve as calibrated sensors to monitor ion concentration, or study the dynamics and flexibility of DDAs. However, DNA nanostructures are difficult to maintain stable in physiological conditions with low salt concentration, and most of ion‐responsive molecules need external ions to trigger. Thus, ion actuation is usually performed in vitro for the detection of ion or controlling simple dynamic behavior of DNA nanostructures.

#### 4.1.2 Light

**Figure 5:**
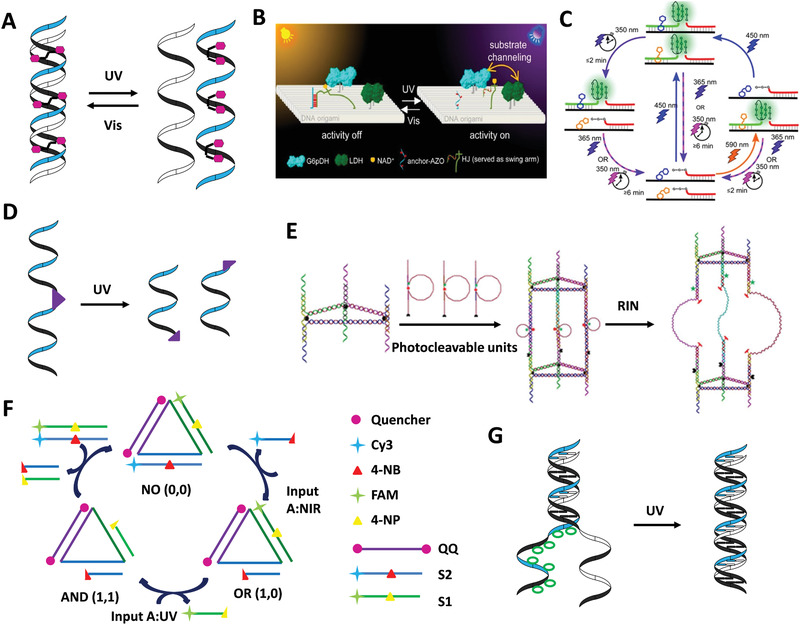
Photoactuation of DDAs. A) Mechanisms of photoresponsive mechanism of Azo‐DNA. B) Light‐driven substrate channeling on DNA origami for precise regulation of enzyme cascade activity. Reproduced with permission.^[^ ^18^ ^]^ Copyright 2018, American Chemical Society. C) Temporal and reversibly control of a DNAzyme system by orthogonal photoswitching. Reproduced with permission.^[^ ^78^ ^]^ Copyright 2018, American Chemical Society. D) Mechanisms of photocleavage. E) Reconfiguration of DNA nanotubes triggered by two‐photon excitation. Reproduced with permission.^[^ ^80^ ^]^ Copyright 2015, Wiley‐VCH Verlag. F) A logic‐gated DNA platform controlled by one‐ and two‐photon excitations. Reproduced with permission.^[^ ^82^ ^]^ Copyright 2016, Wiley‐VCH Verlag. G) Mechanisms of photocaging.

Since azobenzene‐tethered DNA (Azo‐DNA) was synthesized by Asanuma et al. in 2007,^[^ ^70^ ^]^ Azo‐DNA has become a common light‐responsive unit in the DDAs. Azobenzene is a photochromic molecule which presents *trans*‐form in the irradiation of visible light, and turns *cis*‐form when exposed to ultraviolet (UV) light. Therefore, the photoresponsive mechanism of Azo‐DNA is that *trans*‐Azo promotes the formation of dsDNA in vis light, while DNA duplex is dissociated into two single strands promoted by UV‐light irradiation induced isomerization of the *trans*‐Azo to its *cis*‐form (**Figure** **5**A).^[^ ^32^ ^]^ There is a classic 3D reconfigurable chiral plasmonic DNA complex whose conformation change was directly reflected by circular dichroism (CD) spectra.^[^ ^20^ ^]^ Both Azo‐DNA,^[^ ^71^ ^]^ fuel strands,^[^ ^20^ ^]^ aptamers,^[^ ^72^ ^]^ and pH‐sensitive molecules^[^ ^73^ ^]^ have been employed as switch units to control the reconfiguration of the 3D plasmonic object. A DNA rotor functionalized by Azo‐DNA also transformed between perpendicular and parallel states by switching irradiation of UV and visible light.^[^ ^74^ ^]^ Azo‐DNA can also be applied to control the progress of the reaction and regulate the property of molecules. Chen et al. fabricated a light‐induced substrate channeling system on DNA origami nanostructure utilizing Azo‐DNA to control an enzyme cascade reaction (Figure 5B).^[^ ^18^ ^]^ The cofactor, a variable of the reaction, was conjugated to a Holliday junction with one arm containing Azo‐DNA. Owing to the precise positioning ability of DNA origami, the distance between the cofactor and the enzyme cascade was adjusted by the association and disassociation of Azo‐DNAs under the conversion between vis light and UV light, thus inhibiting or activating enzyme cascade activity. Additionally, Dai and Lo employed Azo‐DNA to realize controllable release of cargo that trapped in DNA nanotube.^[^ ^75^ ^]^ In that work, the Azo‐DNA was designed as a hairpin and been incorporated into a DNA nanotube assembled by triangular DNA frameworks. Azo‐DNA hairpins stretched out by UV irradiation, resulting in conformation change of nanotube to release cargo. Thermal stability of photochromic molecule is particularly required for photoregulation systems. The *trans*‐form of molecule is more thermodynamically stable than its *cis*‐form, resulting in the trends of *cis*‐form to be converted into *trans*‐form under ultrahigh‐density irradiation. Arylazopyrazole, a novel Azo derivative, whose *cis*‐form possesses a longer thermal half‐live of 10–1000 days, exhibits near‐quantitative *trans* to *cis* isomerization at 532 and 355 nm.^[^ ^76^ ^]^ Arylazopyrazole‐tethered DNA (Aap‐DNA) shows high thermal stability and conversion efficiency in alternating irradiation at 590 and 365 nm.^[^ ^77^ ^]^ Haydell et al. achieved temporal and reversible control of the catalytic activity of a DNAzyme system through an orthogonal photoswitching system composed of Azo‐DNA and Aap‐DNA (Figure 5C).^[^ ^78^ ^]^

In addition to photoswitching, there are photocleavage and photocaging. Photocleavage is normally achieved by the break of the photocleavable molecules when exposed to light with specific wavelength (Figure 5D).^[^ ^79^ ^]^ Lo and co‐workers incorporated a two‐photon photolabile molecule into DNA strand and formed a photocleavable unit (Figure 5E). The units were connected with two triangular DNA modules to form a photoresponsive DNA nanotube. The photocleavable molecules were cleaved under two‐photon excitation at 800 nm, thus resulting in reconfiguration of the DNA nanotube.^[^ ^80^ ^]^ Furthermore, photocleavage is widely used in the designing of two‐photon fluorescent probe, which can absorb two photons, increase the penetration depth and reduce tissue autofluorescence.^[^ ^81^ ^]^ Lo and co‐workers designed a logic‐gated DNA device, which was controlled by a two‐photon molecule 4‐NB and a one‐photon molecule 2‐NP.^[^ ^82^ ^]^ As shown in Figure 5F, under different inputs (UV and near infrared ray (NIR) irradiation), the logic gate system outputted different fluorescent signals in cancer cells. And, Yang et al. created a two‐photon DNAzyme–gold nanoparticle (AuNP) hybrid probe for intracellular Zn^2+^ imaging.^[^ ^83^ ^]^ Photocaging means inhibiting the activity of the molecules by imprisoning them with photolabile groups (Figure 5G). When exposed to light of specific wavelength, the molecules were released from the “cage” and reactivated. For instance, a closing DNA tweezer whose internal trigger strand were protected by seven photolabile molecules could be opened by removing protected molecules to release the internal trigger strand under UV light.^[^ ^84^ ^]^

Light is an ideal stimulus due to its convenience, cleanness, and fastness. Light‐sensitive components were produced by integrating photochromic molecules into DNA assemblies, and the photosensitivity allows it to be easily triggered. Uncomplicated operation and reversibility of photoactuation make it possible to repeat the reconfiguration of DDAs.^[^ ^85^ ^]^ Nevertheless, there are still several limitations of the photoactuation system. Photoactuation is difficult to apply in vivo because the low penetration depth of UV.^[^ ^86^ ^]^ Red light whose wavelength is longer than UV light is able to effectively penetrate into tissues.^[^ ^87^ ^]^ DNA backbone will be destroyed under high dose UV irradiation, but the photosensitive unit cannot be triggered in low UV dose. Azo can be reduced by endogenous thiols like glutathione (GSH) in cells.^[^ ^87^ ^]^

#### 4.1.3 Temperature

**Figure 6:**
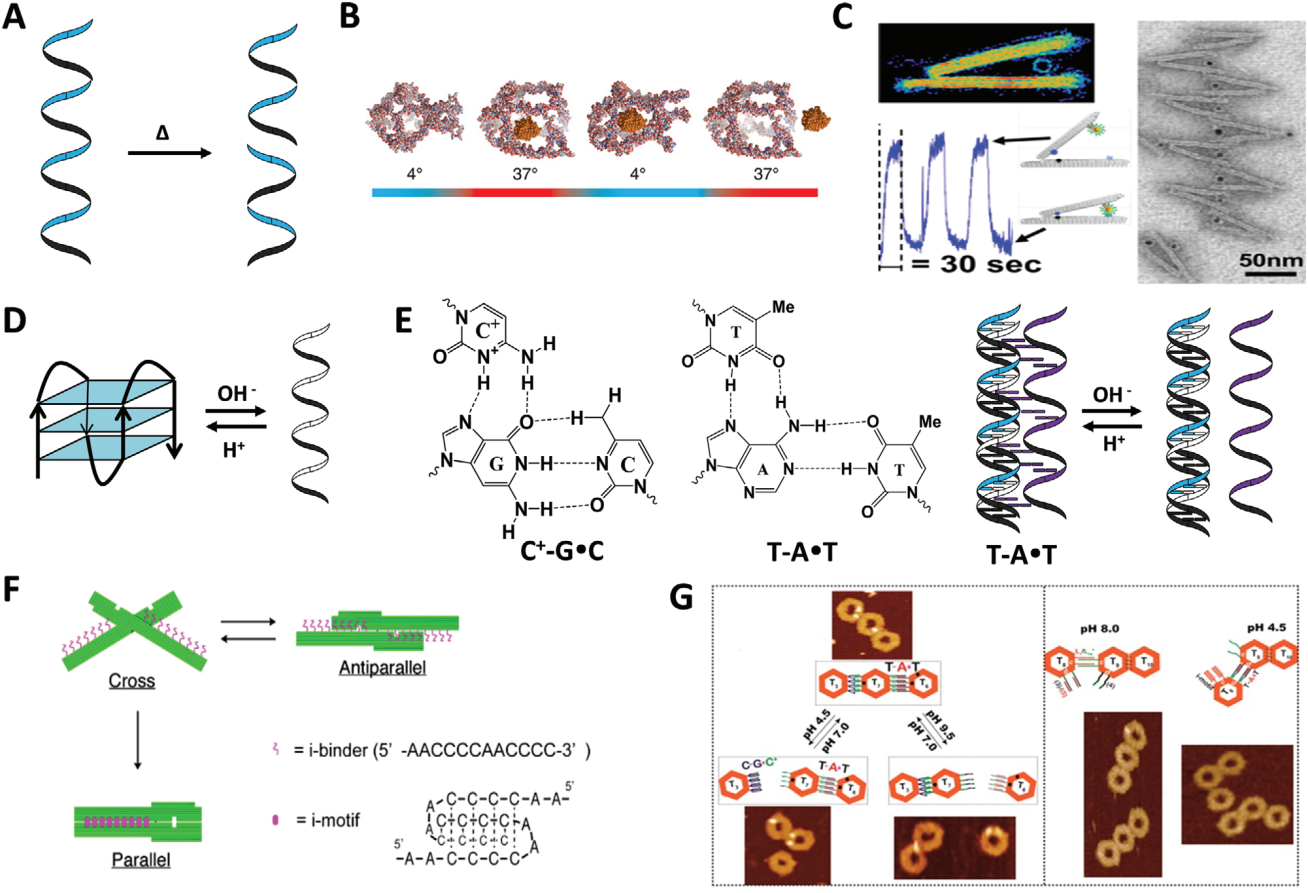
Temperature actuation and proton actuation of DDAs. A) Temperature actuation based on the heat resistance of DNA strand. B) Temperature‐controlled encapsulation and release of cargo in a DNA nanocage. Reproduced with permission.^[^ ^88^ ^]^ Copyright 2013, American Chemical Society. C) A thermally actuated reconfiguration of a hierarchical gold nanoparticles–hinge (AuNPs–hinge) array. Reproduced with permission.^[^ ^90^ ^]^ Copyright 2019, American Chemical Society. D) Proton responsiveness of i‐motif. E) C^+^–G–C and T–A–T DNA triplex. F) A DNA origami pH sensor that can switch between cross, antiparallel, and parallel conformations. Reproduced with permission.^[^ ^97^ ^]^ Copyright 2014, Multidisciplinary Digital Publishing Institute. G) pH‐driven reversible association and dissociation of origami dimer and trimer systems. Reproduced with permission.^[^ ^100^ ^]^ Copyright 2016, American Chemical Society.

Similar to ion actuation, thermal‐responsive DNA assemblies are generally based on thermal‐sensitive molecules (**Figure** **6**A). Thermosensitivity of DNA involves the binding efficiency and structure of oligonucleotides varying with temperature. For example, there is a temperature‐controlled DNA cage that enables encapsulating and releasing of the cargo without any covalent or noncovalent forces (Figure 6B).^[^ ^88^ ^]^ The control unit was a thermal‐sensitive ssDNA, which forms a hairpin structure at 4 °C to trap cargo inside and relaxes at 37 °C, functioning as a rope controlled pocket. A DNA thermometer for monitoring intracellular temperature was based on this principle.^[^ ^89^ ^]^ Taking advantage of differences in heat‐tolerant properties between diffident DNA strands, Tigges et al. presented that the construction of fibrillar DNA superstructures could be mediated by temperature.^[^ ^43^ ^]^ The temperature‐mediated mechanism is that the melting point (*T*_m_) of the connector strands is far below than that of DNA cuboids. When the temperature was between two *T*_m_, the DNA superstructure dissociated and subsequently reassembled. Thermally actuated reconfiguration of a hierarchical AuNPs–hinge array was demonstrated recently (Figure 6C).^[^ ^90^ ^]^ The angle of hinge is adjusted by size of AuNPs and its position in the hinge, thus controlling conformational distribution of the array. The tunable actuation temperature was realized by adjusting the number and length of overhangs in the bottom binding site. After repeated trials, the reconfiguration of the array in response to temperature could be realized within a few seconds.

As for thermal‐sensitive molecule, the principle is also the change in morphology at specific temperature. For example, poly *N*‐isopropylacrylamide (PNIPAM) which forms hydrogel at low temperatures and fractures when the temperature is higher than 32 °C.^[^ ^91^ ^]^ Turek et al. designed a thermal‐responsive DNA origami flexor by incorporating PNIPAM‐modified strands on both sides of the flexure hinge.^[^ ^92^ ^]^ Specifically, when the temperature is above 32 °C, the two arms of origami folded because of hydrophobic interaction of PNIPAM. Once below 32 °C, the PNIPAM rehydrates, thereby opening the origami flexor. Generally, temperature‐responsive mechanism is not often applied in DDAs because of poor heat resistance of DNA. Some thermal‐sensitive DNA strands can be designed for monitoring in vitro temperature changes.

#### 4.1.4 pH

pH‐responsive DDAs are usually incorporated with pH‐responsive units. Cytosine (C)‐rich i‐motif automatically assembles into H^+^‐mediated quadruplex structure in acidic environment, while recovers to single‐strand structure under neutral environment (Figure 6D).^[^ ^93^ ^]^ I‐motif structure usually features with regularly arrangement of the continuous cytosine sequence (C‐tracts) and sequence of adenine (A) and thymidine (T) bases (i‐loops). The sensitivity of i‐motif depends mainly on C‐tracts because the quadruplex is formed by Hoogsteen interactions between four C bases and i‐loops, which adjusts the tension of the quadruplex.^[^ ^94^ ^]^ Furthermore, extensive researches have demonstrated that i‐motif can undergo repeated contractions and expansions.^[^ ^95^ ^]^ DNA triplex formed by ssDNA binded with dsDNA via Hoogsteen bonds is also a pH‐sensitive unit, including C^+^–G·C and T–A·T two types (Figure 6E).^[^ ^96^ ^]^ The former separates in acidic conditions and the latter dissociates in basic conditions. The prominent feature of triplex is that it can be composed of one, two or three separated strands, which diversifies the dynamic behaviors of DDAs.

The mechanism of i‐motif in DDAs can be roughly divided into two types: 1) the hybridization and dehybridization of i‐motif and complementary sequence, and 2) the formation and disintegration of quadruplex between i‐motif fragments. Kuzuya et al. incorporated i‐motif fragments into two levels of a DNA origami plier, achieving controllable switching of the plier in cross (pH 7.0), antiparallel (pH 8.2), and parallel (pH 5.6) conformations (Figure 6F).^[^ ^97^ ^]^ As to DNA triplex, related applications mainly depend on Watson–Crick and Hoogsteen interactions. Kuzyk et al. achieved selective reconfiguration of chiral plasmonic complex with DNA triplex‐functionalized DDAs.^[^ ^73^ ^]^ Right‐handed (RH) and left‐handed (LH) states of the plasmonic complex are controlled by C^+^–G·C and T–A·T triplex, respectively. In mixed system containing RH and LH quasi‐enantiomers, C^+^–G·C and T–A·T triplex showed excellent selectivity with increasing pH values. Besides, given wide responsive range, reversibility and maneuverability, pH‐responsive units greatly enrich origami chemistry that refers to the controllable assembly and disassembly of DNA monomers. Origami chemistry can be controlled by various factors, such as pH‐responsive unit,^[^ ^98^ ^]^ fuel strand,^[^ ^99^ ^]^ DNAzyme,^[^ ^69^ ^]^ etc. Taken pH‐driven system as an example to illustrate origami chemistry.^[^ ^100^ ^]^ As shown in Figure 6G, the trimers were modified by C^+^–G·C and T–A·T, and their states were controlled by adjusting the pH of the solution.

pH‐sensitive DDAs have a wide range of applications, which can monitor changes in pH, and responsive release of drugs from DNA carriers.^[^ ^101^ ^]^ Importantly, the response sensitivity and transition midpoint can be adjusted by sequence changing or proper modification.^[^ ^94^ ^]^ Nevertheless, the application of pH‐regulated system in DDAs is still limited because DNA is prone to denature under extremely acidic or alkalic conditions, which results in the DNA structures to be easily destroyed.

#### 4.1.5 DNA Strands

**Figure 7:**
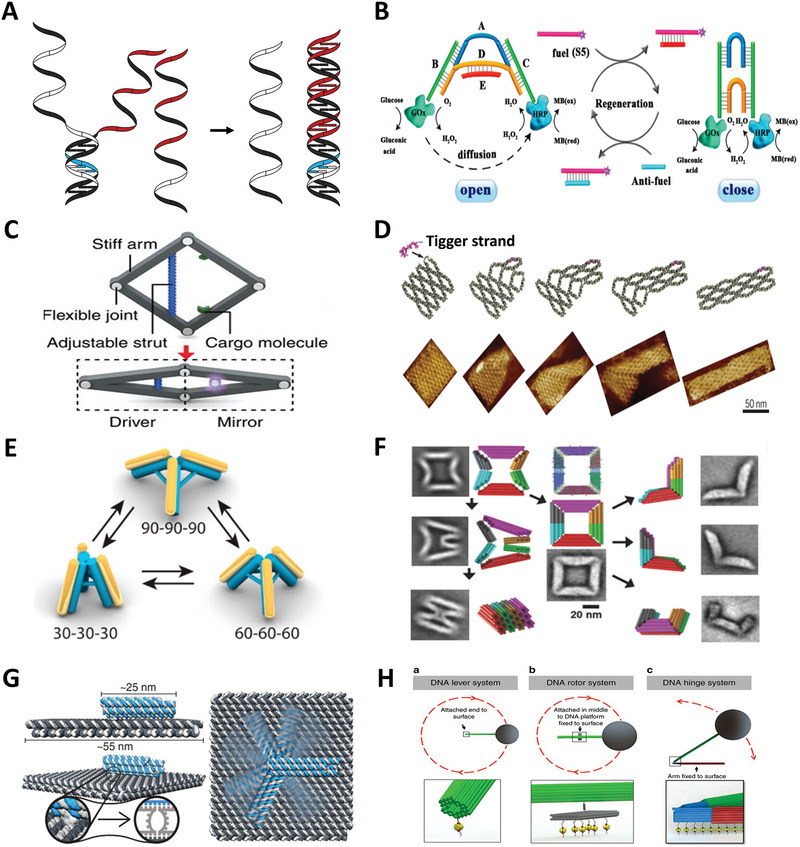
Strand actuation, electric and magnetic actuation. A) Scheme of toehold‐mediated strand displacement reaction. B) The dynamical regulation of the enzyme cascade reaction by a regenerated DNA tweezer. Reproduced with permission.^[^ ^104^ ^]^ Copyright 2018, American Chemical Society. C) A tunable rhombus‐shaped DNA origami nanodevice. Reproduced with permission.^[^ ^106^ ^]^ Copyright 2016, Springer Nature. D) A configurable DNA domino array. Reproduced with permission.^[^ ^109^ ^]^ Copyright 2017, American Association for the Advancement of Science. E) A 3D reconfigurable plasmonic nanostructure. Reproduced with permission.^[^ ^21^ ^]^ Copyright 2017, American Chemical Society. F) A DNA assembly with seven conformations. Reproduced with permission.^[^ ^110^ ^]^ Copyright 2018, Wiley‐VCH Verlag. G) A molecular platform with an integrated rotatable positioning arm. Reproduced with permission.^[^ ^112^ ^]^ Copyright 2018, American Association for the Advancement of Science. H) DNA lever, rotor, and hinge systems actuated by magnetic field. Reproduced with permission.^[^ ^113^ ^]^ Copyright 2018, Springer Nature.

ssDNA has better flexibility and spatial selectivity than dsDNA. ssDNA like i‐motif and hairpin with the characters of contraction and elongation can be used to perform translational movements of nanoscale objects.^[^ ^95^, ^102^ ^]^ The flexibility for pairing and departing of single strand makes toehold‐mediated strand displacement reaction easy to occur (**Figure** **7**A). Therefore, strands actuation is most widely applied in engendering behaviors of complex systems. It usually involves reconfiguring the device by selectively replacing strands (e.g., fuel and antifuel system and lock–key system), driving the device to move via toehold‐mediated strand displacement (e.g., DNA walker), and employing single strand as a probe to detect the target (e.g., DNA probe).^[^ ^103^ ^]^

The fuel and antifuel system is a special reversible type of lock–key system where the locks are functional molecules like i‐motif and DNAzyme, and the keys are corresponding stimulus. In fuel and antifuel system, the lock and key are both oligonucleotides. Kou et al. utilized fuel and antifuel system to design a regenerated DNA tweezer to realize dynamical regulation of enzyme cascade amplification (Figure 7B).^[^ ^104^ ^]^ Two enzymes were individually tethered on the end of two arms of the tweezer, the distance between two enzymes changed as the conversion between open and closed states of the tweezer. And there is a DNA origami vault with an isolated cavity for loading enzyme to control enzyme–substrate reaction via fuel and antifuel systems.^[^ ^105^ ^]^ With sufficient trigger strands, the device could be reversibly opened and closed via toehold‐mediated strand displacement.

DDAs have two possible conformations: open and close states. Multiple configuration transforms or continuous transformation is a challenge for DDAs. While the reconfiguration of DNA nanostructures can be controlled by diverse stimuli, accurate control of multiple conformation conversions is not easy. In this case, the advantages of strand actuation are reflected because the length of single strands can be precisely controlled by adding/deleting bases. Ke's group constructed a rhombus‐shaped DNA origami frame with four flexible joints, which presented opened state due to electrostatic repulsion between arms (Figure 7C).^[^ ^106^ ^]^ They designed an adjustable strut at corresponding symmetrical sites between two arms. The reconfiguration of DNA frame was triggered by the change in length of the strut caused by the hybridization of strut and fuel strands with different length. Utilizing the difference in rigidity between single and double strands, Choi et al. weaved a dynamically controllable DNA accordion rack that is a network of DNA beams linked by multiple flexible joints.^[^ ^107^ ^]^ There are two sticky ends between two DNA beams for hybridizing with lock strands of different lengths, resulting in a local change of the angle between the two beams, thus altering the conformation of the entire network. There is another DNA domino array assembled by antijunction units,^[^ ^108^ ^]^ which could switch between two stable conformations through an unstable opened conformation.^[^ ^109^ ^]^ The reconfiguration was induced by the force generated from the combination of single strand sequence of a antijunction unit and trigger strand (Figure 7D). The transformation path was up to the geometry of the array and the position of the trigger strands. Importantly, the reconfiguration of the domino array can be ended at any designated location by removing or adding units, or by locking strands. The advantage of strand actuation is independence, repeatability and precise regulation capabilities, which makes strand actuation the primary choice for regulating nanostructures with complex dynamic behavior. There is plasmonic DNA origami tripod that can convert in six states. The reconfiguration principle was analogous to rhombus‐shaped DNA frame, but the struts between each two legs were two parallel double helices whose length were adjusted via toehold‐mediated strand displacement reactions (Figure 7E).^[^ ^21^ ^]^ In 2018, inspired by origami, a six‐jointed closed chain was constructed by integrating stiff DNA origami panels with s set of single strands (Figure 7F).^[^ ^110^ ^]^ Through the addition of different fuel and antifuel strands, the structure could be induced to fold along four distinct trajectories, which fully illustrates the flexibility of ssDNA and the induction ability of fuel strands. These nanostructures with distinguishing conformations can be used for loading drugs or imaging agents, and then perform tasks through configuration transformation, and for constructing complex that can perform autonomous movement, such as sliding.^[^ ^111^ ^]^

The affinity between single strands depends on the number of paired bases. And the rigidity of dsDNA can be regulated by correct/wrong base pairing. Above features and unique toehold‐mediated strand displacement behavior enabled the design of DDAs with complex motion. However, despite the ability of programmatically and orthogonally controlling multiple elements, an obvious disadvantage of strand actuation is that the trigger strands must be added externally. And the trigger strands are usually added in high molar excess to achieve suitable kinetics, thereby causing waste. Also, the time needed for triggering is long.

#### 4.1.6 Electric and Magnetic Field

Electric and magnetic actuations own improvements in operation speed, switching efficiency, and the ability of controlling nanoscale motion. Kopperger et al. constructed a clock analogues DNA‐based molecular device with a robotic arm serving as hour hand whose movement was manipulated by an electric field (Figure 7G).^[^ ^112^ ^]^ The mechanical arm was connected to DNA origami platform via a flexible joint that gives the arm a quite large range of rotation. The negatively charged DNA nanostructure was placed in an electric field, so the voltage can be applied to arbitrarily control the direction of the arm. As for magnetic actuation, it needs to be implemented with magnetic nanoparticles. In a magnetic field‐controlled system, a superparamagnetic bead affixed to one end of the mechanical lever was the inducer for magnetic actuation. The magnetic actuation was demonstrated both in continuous rotation of a rotor system and oscillating opening and closing of a hinge system (Figure 7H).^[^ ^113^ ^]^ There is another rotor that can perform random Brownian rotary motion without any energy support.^[^ ^114^ ^]^ Directional rotation of it could be realized by the addition of electric or magnetic fields. External physical actuation allows direct manipulation of DNA nanostructures with nanoscale precision and short response time, which are more sensitive and faster than chemical and biological actuations. Further, electric and magnetic actuations allow multiple components to be driven simultaneously. Exogenous stimuli in DDAs and related applications are summarized in **Table** **2**.

**Table 2:** Exogenous stimuli in DDAs and related applications

Stimulus	Functional components	Applications	Refs.
Ions	Short DNA overhang	Salt concentration monitoring	^[^ ^61^ ^]^
	Ion‐dependent G‐quadruplex	Detection of ions	^[^ ^65^ ^]^
	Ion‐dependent DNAzyme	Detection of ions	^[^ ^69^ ^]^
Light	Azo‐DNA	Construction of reconfigurable chiral plasmonic object	^[^ ^71^ ^]^
		Controlling the enzyme cascade reaction	^[^ ^18^ ^]^
		Controllable release of cargo	^[^ ^85^ ^]^
	Aap‐DNA	Controlling the catalytic activity of DNAzyme	^[^ ^78^ ^]^
	Photolabile molecule	Controllable release of cargo	^[^ ^115^ ^]^
Temperature	Short DNA strand	Controllable release of cargo	^[^ ^88^ ^]^
		Monitoring intracellular temperature	^[^ ^89^ ^]^
	Thermal‐sensitive molecule	Controlling catalytic reaction	^[^ ^92^ ^]^
pH	I‐motif	pH monitoring	^[^ ^97^ ^]^
	DNA triplex	Construction of reconfigurable chiral plasmonic object	^[^ ^73^ ^]^
DNA strand	Fuel and antifuel strand system	Regulation of enzyme cascade amplification	^[^ ^104^ ^]^
		Controlling enzyme–substrate reaction	^[^ ^105^ ^]^
		Construction of complex with autonomous movement	^[^ ^111^ ^]^
Electric field	Negatively charged DNA nanostructure	Construction of intelligent system	^[^ ^112^ ^]^
Magnetic field	Magnetic nanoparticles	/	^[^ ^113^ ^]^

### 4.2 Endogenous Stimuli

Except for pH actuation, other aforementioned actuations are difficult to achieve in vivo because of low sensitivity or the addition of exogenous stimuli. As for the application of DDAs in vivo, it is more feasible and convenient to design the switching units according to endogenous stimulus, particularly the different microenvironments between abnormal and normal cells microenvironment.

#### 4.2.1 GSH

**Figure 8:**
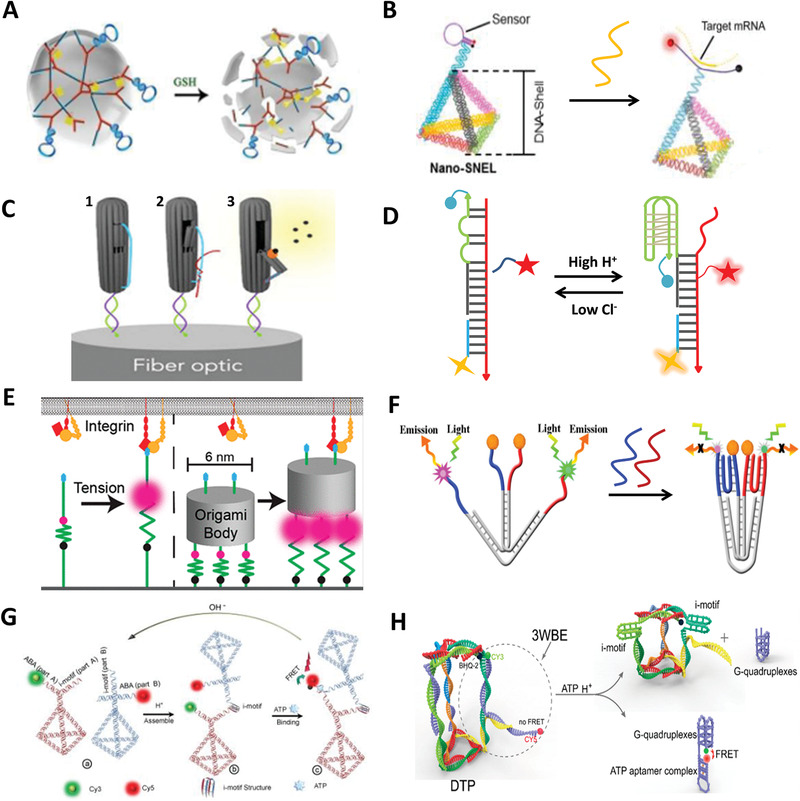
Exogenous stimuli. A) GSH‐responsive DNA hydrogels. Reproduced with permission.^[^ ^120^ ^]^ Copyright 2015, American Chemical Society. B) Snail‐simulated DNA nanosensor for real‐time detection of mRNA in living cells. Reproduced with permission.^[^ ^123^ ^]^ Copyright 2015, American Chemical Society. C) DNA origami‐optical fiber genosensor for target DNA detection. Reproduced with permission.^[^ ^127^ ^]^ Copyright 2017, Elsevier. D) A double‐stimulated DNA reporter that can quantitatively image pH and Cl^−^ simultaneously in lysosomes. Reproduced with permission.^[^ ^132^ ^]^ Copyright 2018, Spring Nature. E) A DNA origami tension probe for the measurement of the piconewton forces generated by living cells. Reproduced with permission.^[^ ^136^ ^]^ Copyright 2018, American Chemical Society. F) A dual‐functional DNA tweezer for simultaneous detection of microRNA and protein. Reproduced with permission.^[^ ^144^ ^]^ Copyright 2019, American Chemical Society. G) The formation of DNA tetrahedral dimer. Reproduced with permission.^[^ ^149^ ^]^ Copyright 2019, Wiley‐VCH Verlag. H) Working principle of the logic triangular prism. Reproduced with permission.^[^ ^150^ ^]^ Copyright 2019, American Chemical Society.

GSH is a biological tripeptide consisting of glutamic acid, cysteine, and glycine with multiple biological functions. The concentration of GSH in the living cells is hundred times higher than that in extracellular space, while the level of GSH in tumor cells is much higher than that in healthy tissues.^[^ ^116^ ^]^ The GSH‐responsive DDSs are based on the reduction of specific chemical bonds such as disulfide,^[^ ^117^ ^]^ diselenide,^[^ ^118^ ^]^ thioether,^[^ ^119^ ^]^ etc., by intracellular GSH. In 2015, Li et al. synthesized GSH‐responsive DNA hydrogels for gene therapy.^[^ ^120^ ^]^ Disulfide bonds were inserted in DNA strands, GSH induced disulfide bond breakage and caused disintegration of hydrogel, thus releasing intracellular antisense oligonucleotides (**Figure** **8**A). Besides, Jiang et al. individually fabricated plasmonic rhombus‐shaped DNA origami templates, which responded to GSH.^[^ ^121^ ^]^ The structural transformation of GSH‐stimulated system relied on reduction of disulfide bonds in the controller strands between two triangular DNA plates. In fact, in addition to being drug loading vehicles, GSH‐responsive DDAs are also excellent tools for detecting GSH levels in diseased cells or subcellular organelles.

#### 4.2.2 Endogenous Nucleic Acids

Endogenous nucleic acids are closely related to physiological activities of cells. Messenger ribonucleic acid (mRNA) is responsible for genetic information transport, ribosomal RNA (rRNA) for protein synthesis, microRNAs for various signal pathway adjustment, etc. In cancer diagnosis, a vital criterion is the abnormal expression of genes in living cells. The expression level of tumor‐related nucleic acids reflects related information about cancer progression and prognosis. Most endogenous nucleic acids have a short half‐life and are constantly being synthesized and degraded, which requires that the probes for nucleic acids detection achieve higher cellular uptake efficiency and capabilities of resistance to DNAase degradation. Tetrahedral DNA nanostructure (TDN) has been widely employed in nucleic acids detection due to its simple preparation and excellent stability.^[^ ^122^ ^]^ Inspired by the structure of snail's tentacles, Tay et al. designed a TDN with antennas for mRNA detection (Figure 8B).^[^ ^123^ ^]^ The antennas was a hairpin modified with two terminal fluorophores. The two fluorophores separated when the target mRNA molecule hybridized with the hairpin, resulting in an enhancement in the fluorescent signal. In another TDN probe for mRNA detection, the hairpin was embedded in one edge of the TDN.^[^ ^124^ ^]^ The reconfiguration from contraction to extension of TDN caused by the hybridization of the hairpin and mRNA was converted into variation in fluorescence intensity. In addition, TDN probes achieved rapid detection of dengue virus RNA sequence^[^ ^125^ ^]^ and endogenous DNA.^[^ ^126^ ^]^ A electrochemical TDN sensor showed quantitative response to the target DNA in nanomolar precision of measurement.^[^ ^126^ ^]^ DNA assemblies with small size and simple configuration are more used for intracellular detection. While in extracellular detection, DNA origami with better biological stability is preferred. Recently, a DNA nanorobot for detecting tobacco mosaic virus (TMV) nucleocapsid gene was developed (Figure 8C).^[^ ^127^ ^]^ The space capsule‐shaped DNA nanorobot has a small switchable flap with a controller strand whose two ends were separately fixed to the top of flap and the surface of the capsule. The flap was opened due to the reduced length of controller via hybridizing with target DNA. The signal transduction was realized by exposing DNAzyme analogue to catalyze H_2_O_2_‐mediated oxidation of luminol agent, resulting in chemiluminescent signals. The open of the door requires precise control of the length to avoid half‐opened state.

#### 4.2.3 pH

The pH characteristics differs in different organs, such as the neutral environment of normal tissue (around pH 7.4), slightly acidic microenvironment surrounding cancer cells (pH 6.5–7.2), and acidic condition of lysosome and exosomes (about 5.0).^[^ ^128^ ^]^ Furthermore, the abnormal pH levels of organelles are related to some diseases, such as alkaline lysosomal pH (up to 6.1) in some lysosomal storage diseases.^[^ ^129^ ^]^ Therefore, pH‐responsive DDAs have a wide range of applications. Both i‐motif and C^+^–G·C triplex were used to be incorporated into DNA tweezers that anchored on the surface of cell membrane for real‐time monitoring of extracellular pH change.^[^ ^130^, ^131^ ^]^ In the virtue of quantitative imaging and lysosome addressability function of DNA nanodevices, Leung et al. designed a double‐stimulated DNA reporter that can quantitatively image pH and Cl^−^ simultaneously in the lysosomes (Figure 8D).^[^ ^132^ ^]^ Via analyzing two ion measurement (2‐IM) profiling of lysosome, the content of H^+^ and Cl^−^ in lysosome could be reflected with high efficiency and accuracy. 2‐IM profiling produced by the DNA reporter not only can be used to discriminate lysosome chemotypes, but also is a promising method for tracking some disease progression that involves lysosome variation.

#### 4.2.4 Protein

Protein actuation usually involves receptor–ligand interaction such as folic acid and the folate receptor‐*α*,^[^ ^133^ ^]^ transferrin and the transferrin receptor,^[^ ^134^ ^]^ hyaluronic acid and the cluster of differentiation 44 (CD44),^[^ ^135^ ^]^ etc. Utilizing the variation in length caused by the transition between single strand and double strand, Dutta et al. designed a DNA origami tension probe to measure the piconewton forces generated by the interaction between integrin and adhesive peptide (cRGDfk) (Figure 8E).^[^ ^136^ ^]^ The probe consisted of three parts: a ligand‐binding domain, an origami intermediate, and a force sensing domain containing a tunable number of extensible hairpins tagged with a fluorophore–quencher pair near the base of its stem. The hairpin unfolded when ligand bound to receptor on the cell surface, thereby separating the fluorophore and quencher producing an increase in fluorescence. To some extent, the unfolding force of the probe measured by changing the number of hairpins reflects receptor traction forces in living cells. However, given the rigidity of six‐helix bundle of origami, the force generated by the combination of receptor and ligand transmitted through the ligand was not averagely distributed to each hairpin. In receptor–ligand interactions, the biomarker–aptamer interaction is a unique interaction, in which, biomarkers are overexpressed biomolecules, and a plenty of biomarkers can be specially recognized by aptamer.^[^ ^137^ ^]^ Aptamer is a small segment of oligonucleotide sequence screened by systematic evolution of ligands by exponential enrichment (SELEX) technique,^[^ ^138^ ^]^ showing high binding affinity and specificity toward various targets by forming various complementary structures,^[^ ^139^ ^]^ such as specific recognition between nucleolin and AS1411,^[^ ^140^ ^]^ prostaglandin specific membrane antigen (PSMA) and A10,^[^ ^141^ ^]^ and epidermal growth factor receptor (EGFR) and CL4.^[^ ^142^ ^]^ Aptamer‐functionalized DDAs have become versatile platforms particularly for targeted drug delivery and biosensing.^[^ ^22^, ^143^ ^]^ In a dual‐detection system, miRNA 21 recognition sequence and mucin 1 (MUC1) aptamer hybridized with complementary sequence R1 and R2 were conjugated on the surface of Au@Fe_3_O_4_. The W‐type dual‐functional DNA tweezers were responsible for detection. When the targets were recognized, R1 and R2 were released to combine with the tweezer, leading to the deformation of the tweezers and noticeable changes in fluorescent signal (Figure 8F).^[^ ^144^ ^]^ As functional nucleic acid with powerful recognition ability, aptamer can be directly integrated into DDAs without exogenous linkers, which plays a vital role in the application of DNA assemblies.

#### 4.2.5 Adenosine Triphosphate (ATP)

The ATP concentration among intercellular, extracellular, and lysosomal condition varies with different situations. For example, the lysosomal ATP level in cells during starvation and hypoxia is far lower than that in the normal cells. In ATP‐triggered system, mostly used probes are ATP aptamers.^[^ ^145^ ^]^ Aptamer that can bind to the target molecule after being split into two fragments, which called split aptamer.^[^ ^146^ ^]^ Unique structural features and diversity of ATP aptamers make ATP a popular trigger molecule in analysis, detection, and DDSs.^[^ ^147^ ^]^ The signal conversion of a DNA “origami” traffic light was controlled by split ATP aptamers.^[^ ^148^ ^]^ According to the characteristics of low pH and high ATP concentration in lysosomes, Peng et al. constructed a logic framework nucleic acid nanoplatform consisted of two TDNs with different branching vertices, both carrying a split i‐motif sequence and a split ATP aptamer (Figure 8G).^[^ ^149^ ^]^ The two monomeric TDNs were taken up by cells and eventually responded to H^+^ and ATP in lysosome to form a heterodimeric architecture, resulting in a fluorescent signal transition. In addition, Peng's group incorporated a dual‐responsive component (DRC) into DNA triangular prism (DTP) for lysosomal imaging (Figure 8H).^[^ ^150^ ^]^ The DRC was composed of Cy5‐labled ATP aptamer and Cy3‐labled K^+^ G‐quadruplex aptamer, which was incorporated into DTP by the hybridization of i‐motif and G‐quadruplex. When DTP entered lysosomes, i‐motif sequences responded to H^+^ leading to deformation of DTP, thus releasing DRC from DTP. In the presence of cellular K^+^ and ATP, DRC automatically folded into a reporter structure, which allowed the two fluorophores close to each other, leading to the occurrence of FRET.

DDAs that respond to endogenous stimulus are commonly applied in disease treatment and diagnosis. The process of endogenous stimulus actuation is totally automatic, while the stability of DNA nanostructures, and the sensitivity and specificity of responsive units are essential. To some extent, DNA assemblies with relatively simple construction have greater flexibility and are superior to bulky DNA assemblies in detection. Structural flexibility and responsive sensitivity make multitarget simultaneously detection possible.^[^ ^144^ ^]^ Endogenous stimuli in DDAs and related applications are summarized in **Table** **3**.

**Table 3:** Endogenous stimuli in DDAs and related applications

Stimuli	Functional components	Applications	Refs.
GSH	Disulfide bond	Gene therapy	^[^ ^120^ ^]^
pH	I‐motif	Extracellular pH monitoring	^[^ ^131^ ^]^
		Quantitatively imaging of lysosomal pH	^[^ ^132^ ^]^
	DNA triplex	Extracellular pH monitoring	^[^ ^130^ ^]^
Endogenous nucleic acids	Corresponding complementary sequence	Detection of mRNA	^[^ ^122^, ^123^, ^124^ ^]^
		Detection of dengue virus RNA sequence	^[^ ^125^ ^]^
		Detection of target DNA	^[^ ^126^ ^]^
		TMV nucleocapsid gene	^[^ ^127^ ^]^
		MicroRNA	^[^ ^144^ ^]^
Protein	Hairpin	Measurement of the piconewton forces of ligand–receptor interaction	^[^ ^136^ ^]^
	Aptamer	Detection of protein	^[^ ^144^ ^]^
ATP	ATP aptamer	Detection of ATP level	^[^ ^151^ ^]^
		Lysosomal imaging	^[^ ^150^ ^]^

## 5 Special Dynamic Behaviors of DDAs in Biomedical Applications

### 5.1 DNA Walkers

Just as the name implies, DNA walker means DNA device that can walk on multifarious tracks, such as carbon nanotube,^[^ ^152^ ^]^ metal nanoparticle,^[^ ^153^ ^]^ oligonucleotides,^[^ ^154^ ^]^ DNA origami tile,^[^ ^155^ ^]^ etc. The fundamental principle of DNA walker is toehold‐mediated strand displacement mentioned before. And movement of the walker usually requires the aid of DNAzymes,^[^ ^156^ ^]^ nucleases,^[^ ^153^ ^]^ fuel strand,^[^ ^157^ ^]^ etc.

**Figure 9:**
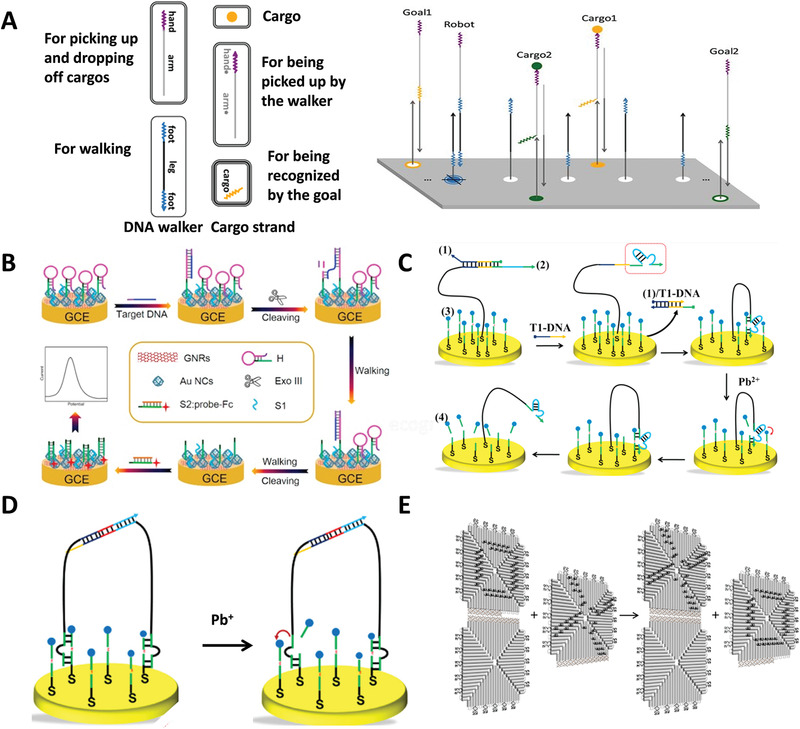
DNA walkers. A) Schematic diagram of a cargo‐sorting system. Reproduced with permission.^[^ ^158^ ^]^ Copyright 2017, American Association for the Advancement of Science. B) A stochastic DNA walking electrochemical biosensor. Reproduced with permission.^[^ ^160^ ^]^ Copyright 2018, Elsevier. C) A DNAzyme‐functionalized ssDNA walker for DNA detection. Reproduced with permission.^[^ ^161^ ^]^ Copyright 2018, Elsevier. D) A DNAzyme‐functionalized dsDNA walker. Reproduced with permission.^[^ ^162^ ^]^ Copyright 2018, Elsevier. E) Scheme of DNA tile displacement. Reproduced with permission.^[^ ^167^ ^]^ Copyright 2018, Springer Nature.

In terms of the number of feet, DNA walker can be classified into three types: inchworm walker, bipedal walker, and spider walker. The structure of the inchworm walker is the simplest, usually a single strand, therefore inchworm walker is flexible and easy to operate. An inchworm walker designed by Thubagere and co‐workers was able to perform cargo sorting at the molecular level.^[^ ^158^ ^]^ As shown in **Figure** **9**A, the walker was composed of upper part for picking up and dropping off cargo strand and lower part for walking. Cargo strand consisted of molecular‐conjugated sequence for being picked up by walker and cargo sequence for being recognized by the sequence of goal strand. Such precise and complex operations are attributed to unprecedented addressability with sub‐nanometer accuracy of DNA origami, which is a powerful platform for DNA walker to perform sophisticated mission. The DNA walker can be triggered by target strands.^[^ ^159^, ^160^ ^]^ Feng et al. designed an electrochemical biosensor triggered by target strands (Figure 9B).^[^ ^160^ ^]^ Track strands immobilized on electron via capture strands were stem‐loop structural hairpin. The target strand bonded to the hairpin first, and then moved to the next with the aid of enzyme until all capture strands were exposed. The current was produced when ferrocene‐labeled probes hybridized with the captured sequences.

Bipedal walker is single strand or double strand structure with one leg fixed on tracks and the other leg for performing taskes. Wang's group individually designed a ssDNA and a dsDNA walker for detecting DNA strands.^[^ ^161^, ^162^ ^]^ The ssDNA walker whose one end was fixed on Au electrode and another end containing DNAzyme sequence was inhabited by locking strand initially (Figure 9C).^[^ ^161^ ^]^ DNAzyme sequence was activated after the hybridization of locking strand and target strand, thus cleaving the signal strands immobilized on Au electrode releasing electroactive labels. In contrast, the dsDNA walker was formed after the pre‐DNA walker being triggered by target strand via strand displacement. Subsequent signal output was the same as ssDNA walker (Figure 9D).^[^ ^162^ ^]^ Moreover, a DNAzyme walker that successfully in response to microRNA in living cells has been reported.^[^ ^163^ ^]^ Recently, Li et al. designed a smarter strand detecting system based on isothermal strand‐displacement polymerase reaction (ISDPR).^[^ ^164^ ^]^ The walker was combined with partial sequence of DNA hairpin anchored on a magnet microparticle, thereby exposing stem of hairpin to hybridize with biotin‐labeled primer. In the presence of polymerase, the interaction between DNA hairpin and gradually extending primer continuously strengthen, forcing target strand transfer to another DNA hairpin. It was demonstrated that the detection method was capable of DNA sensing in human serum.

Spider walker refers to walker with more than two legs.^[^ ^165^, ^166^ ^]^ Classic spider walker can autonomously execute sequences instructions such as “start,” “follow,” “turn,” and “stop” on appropriately designed DNA origami.^[^ ^165^ ^]^ A multilegged DNA walker achieved precise assembly of complex object on origami platform. Compared to two walkers above, spider walker is much less because each movement of it was driven by the addition of different fuel strands, which is sophisticated and hard to control.^[^ ^166^ ^]^ In addition, DNA tile displacement was demonstrated recently (Figure 9E).^[^ ^167^ ^]^ The toehold and branch migration domains of tile displacement consist of a set of edge staples instead of a part of ssDNA fragment. Tile displacement expanded new means for controlling structural reconfiguration of DNA origami assemblies, making it possible to change any desired structural components at the appropriate time.

The strong migration capacity and excellent flexibility make DNA walkers multifunctional tools for quantification and detection of biomolecules, and the photoactuation and chemical energy actuation of DNA walker were also realized recently.^[^ ^168^, ^169^ ^]^ In the design of DNA walker system, the key is the length of footholds, which determines not only the success of the movement but also the walking speed. In the case of correct execution of the command, the walking speed is inversely proportional to the length of the footholds.^[^ ^170^ ^]^ Inchworm walkers and bipedal DNA walkers are highly flexible, which can take multiple steps autonomously once being activated. At the same time, they have very limited bioanalytical applications for signal amplification and complex assembly due to low processivity. The application range of multilegged DNA walkers is broad, but their movement usually requires external intervention. Additionally, DNA walkers with relatively simple structures are prone to be destroyed in vivo. More information and applications of DNA walkers are summarized in **Table** **4**.

**Table 4:** Summary of DNA walkers in biomedical applications

DNA walkers	Walking platforms	Applications	Refs.
Inchworm walker	Carbon nanotube	In situ cancer cell growth inhibition	^[^ ^152^ ^]^
		Molecular transport	^[^ ^171^ ^]^
	Oligonucleotides	/	^[^ ^154^ ^]^
	Glassy carbon electrode	DNA detection	^[^ ^160^ ^]^
	Au electrode	Detection of breast cancer cell	^[^ ^172^ ^]^
	SiO_2_	Detection of miRNAs	^[^ ^173^ ^]^
	Origami tile	Signal amplification	^[^ ^159^ ^]^
		Cargo sorter	^[^ ^158^ ^]^
	Live cell membrane	Monitoring dynamic and transient membrane lipid encounters	^[^ ^174^ ^]^
Bipedal walker	Au electrode	Detection of nucleic acid	^[^ ^161^, ^162^, ^175^ ^]^
		Detection of microRNA	^[^ ^176^ ^]^
		Detection of protein	^[^ ^177^ ^]^
	AuNP	Sensing nucleic acid	^[^ ^178^ ^]^
		Cellular analysis and imaging	^[^ ^163^, ^179^ ^]^
	Magnetic nanoparticle	DNA detection	^[^ ^164^ ^]^
	Glass slide	Quantification of Nucleic Acid	^[^ ^180^ ^]^
	Oligonucleotide	Molecular transport	^[^ ^181^ ^]^
	Origami nanostructure	/	^[^ ^169^ ^]^
Spider walker	Origami nanostructure	Executing predetermined instructions	^[^ ^165^ ^]^
		Cargo assembler	^[^ ^166^ ^]^

### 5.2 DNA Hydrogels

DNA hydrogels refer to hydrogels prepared by physical entanglement or chemical connection of DNA strands. According to composition, DNA hydrogels can be divided into pure DNA hydrogels and hybrid. The first hybrid DNA hydrogel was created by Nagahara and Matsuda in 1996.^[^ ^182^ ^]^ Hybrid hydrogels were composed of DNA‐modified copolymers, and hydrogels formation was induced by the hybridization of complementary strands. There are two methods to prepare pure DNA hydrogels: 1) The elongation of DNA. This method involves elongating DNA strands by rolling circle amplification (RCA) or multiprimed chain amplification (MCA) first, and then weaving them into a hydrogel.^[^ ^183^ ^]^ 2) The combination of branched DNA molecules. In a pure DNA hydrogel system constructed by Luo and co‐workers, the branched DNA blocks were designed with palindromic complementary sticky end, and the formation was catalyzed by T4 DNA ligase.^[^ ^184^ ^]^ Compared to conventional hydrogels, the deformation of DNA hydrogels can be controlled by a variety of stimuli. DNA hydrogels with dynamic behavior are called intelligent DNA hydrogels here.

**Figure 10:**
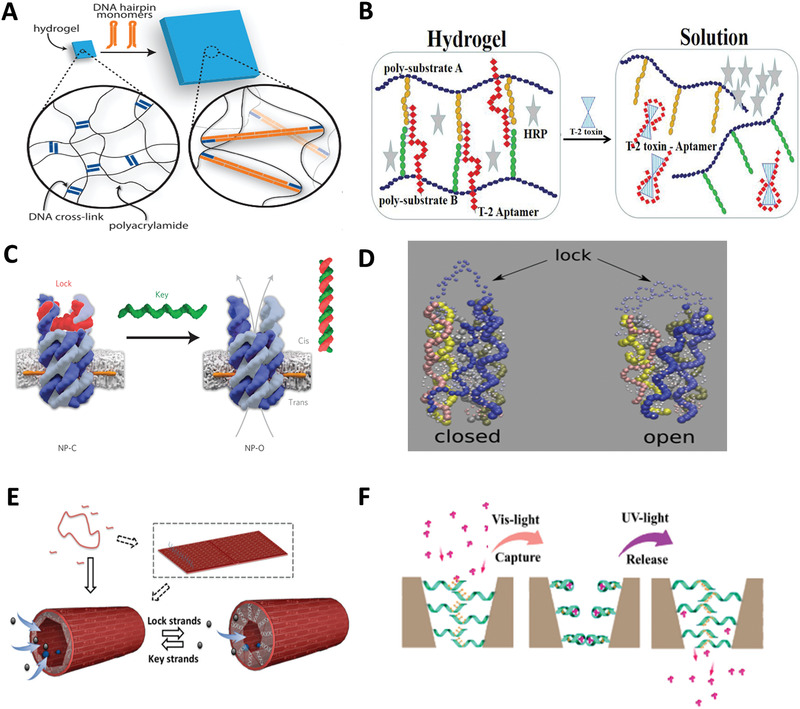
Intelligent DNA hydrogels and dynamic DNA nanochannels. A) Swelling of DNA hydrogels. Reproduced with permission.^[^ ^186^ ^]^ Copyright 2017. American Association for the Advancement of Science. B) Detection of T‐2 toxin by a dynamic DNA hydrogel. Reproduced with permission.^[^ ^206^ ^]^ Copyright 2020, Elsevier. C) The dynamic nanochannel with a lock–key system. Reproduced with permission.^[^ ^199^ ^]^ Copyright 2016, Nature Publishing Group. D) A tensegrity‐driven DNA nanochannel. Reproduced with permission.^[^ ^200^ ^]^ Copyright 2017, The Royal Society of Chemistry. E) A switchable DNA origami nanochannel with multiple lock–key systems. Reproduced with permission.^[^ ^201^ ^]^ Copyright 2016, The Royal Society of Chemistry. F) A photoresponsive nanochannel for capture–release–transport of ATP. Reproduced with permission.^[^ ^204^ ^]^ Copyright 2018, American Chemical Society.

Intelligent DNA hydrogels that respond to pH,^[^ ^185^ ^]^ DNA strand,^[^ ^186^ ^]^ protein,^[^ ^187^ ^]^ light,^[^ ^188^ ^]^ etc., have been developed, and showed great prospects in the fields of biomedicine, such as detection of ion^[^ ^189^ ^]^ and cancer cells,^[^ ^190^ ^]^ drug delivery,^[^ ^120^ ^]^ etc. For instance, Schulman's group realized 100‐fold volumetric expansion of DNA hybrid hydrogel by extending the length of DNAs crosslinks between polymers (**Figure** **10**A).^[^ ^186^ ^]^ The mechanism was similar to toehold‐mediated strand displacement reaction. The toehold sequences were designed in the DNA linkers to induce the hybridization of two hairpins and linkers, thereby extending the crosslinks in the hydrogel. Based on that work, they further developed molecular controllers for amplifying stimuli and releasing signals to indicate the response.^[^ ^191^ ^]^ Recently, Sun et al. developed a DNA hydrogel for detecting the T‐2 toxin (Figure 10B). In that system, the hydrogels was formed by the hybridization of T‐2 toxin aptamer and DNA linkers of polymers, thereby encapsulating the horseradish peroxidase (HRP). When the aptamer bound to T‐2 toxin, the hydrogel collapsed and released the HRP, thus catalyzing the reaction of H_2_O_2_ and KI, leading to the generation of large amounts of I_2_. Afterward, the gold nanorods (AuNRs) were corroded by I_2_ in the presence of a high concentration of cetyltrimethyl ammonium bromide (CTAB), resulting in the variation of size and color of AuNRs. In addition, Mao and co‐workers reported a DNAzyme‐functionalized hydrogel for circulating tumor DNA (ctDNA) detection.^[^ ^192^ ^]^ The target ctDNA was designed as a trigger for inducing RCA, thus preparing DNA hydrogel with hemin‐dependent G‐rich sequences. After the addition of hemin, G‐quadruplex/hemin complex was formed functioning as DNAzyme to catalyze color reaction. By controlling the concentration of hemin, the ctDNA could be qualitatively and quantitatively analyzed.

The majority of hydrogels is chemically linked and therefore exhibiting high stability, while DNA hydrogels exhibit excellent thixotropic properties because they are mainly stabilized by noncovalent interactions. Excellent thixotropic property makes DNA hydrogels show strong advantages in the preparation of injectable hydrogels,^[^ ^193^ ^]^ while, incompact structure makes DNA hydrogels with low storage modulus, and precise control of intelligent DNA hydrogel state transition requires rigorous design and trial.

### 5.3 DNA Nanochannels

Nanopore is a technique for detecting and analyzing single molecules in solution, such as polymer, DNA and RNA sequencing, folding of proteins, polypeptide, etc. In nanopore single molecule sensor device, the electrolyte solution of nanopore is isolated into two separated parts by external voltage. Once single molecule crosses the nanopore, the current can be detected due to the reduced flow of ion. DNA nanostructures applied to nanopore were called DNA nanochannels, which were applied in two ways: the combination of DNA nanostructures and solid state nanopore,^[^ ^194^ ^]^ and inserting DNA nanochannels into lipid membrane simulating natural membrane channels.^[^ ^195^, ^196^ ^]^ As for the inserting, DNA nanochannels overcome the electrostatic repulsion of negatively charged DNA nanostructures and cell membrane via hydrophobic lipid anchors. Hydrophobic belt,^[^ ^196^ ^]^ cholesterol,^[^ ^197^ ^]^ tetraphenyl porphyrin tag anchors,^[^ ^198^ ^]^ etc., have been used.

In 2016, Burns et al. created a relatively simple DNA nanochannel with seven DNA strands, serving as a valve of lipid membrane.^[^ ^199^ ^]^ The nanochannel was inserted into the membrane by cholesterol‐based membrane anchors outside it, and the entrance of cargo was controlled by a lock–key system of the nanochannel (Figure 10C). A similar DNA nanochannel with a 30 nt lock strand was designed by Mendoza et al. (Figure 10D).^[^ ^200^ ^]^ The opening and closing of the nanochannel depended on the difference in mechanical tension between ssDNA and dsDNA. In the presence of key strands, the nanochannel was opened by the tension produced by hybridization of the lock strand. Wangs’ group reported an intelligent DNA nanochannel formed by rolling up a DNA origami blanket with 11 overhangs (Figure 10E).^[^ ^201^ ^]^ The nanochannel switched on and off by adding and removing key strands, which can be used to detect multiple strands simultaneously. In addition to pure DNA nanochannels, the incorporation of nucleic acid molecules into other biomimetic nanochannels is also common.^[^ ^202^ ^]^ Azo‐DNA strands have been used to modify the interior of a polyethylene terephthalate (PET) channel, thereby forming a light‐stimulated nanochannel to control molecules transport.^[^ ^203^ ^]^ A similar photoresponsive nanochannel achieved capture–release–transport of ATP by light‐responsive ATP aptamers modified with Azo‐groups (Figure 10F).^[^ ^204^ ^]^ ATP molecules were captured by aptamers under the irradiation with vis light and then released upon exposure to UV light.

The majority of DNA nanochannels was static, however, developing dynamic DNA nanochannels to control the transportation of substance under controllable responsiveness is the most intriguing direction. How to combine DNA nanochannels with natural proteins and other components to construct multifunctional membrane channels that rivals natural ones is another arduous task. Seifert's group reported a DNA nanochannel that showed tow voltage‐dependent conductance states.^[^ ^205^ ^]^ Nanochannels exhibited open state and stable high‐conductance level under low transmembrane voltages, and naturally closed state corresponding to high transmembrane voltages. The relationship between DNA nanochannel and voltage need to be further studied.

### 5.4 DNA‐PAINT

**Figure 11:**
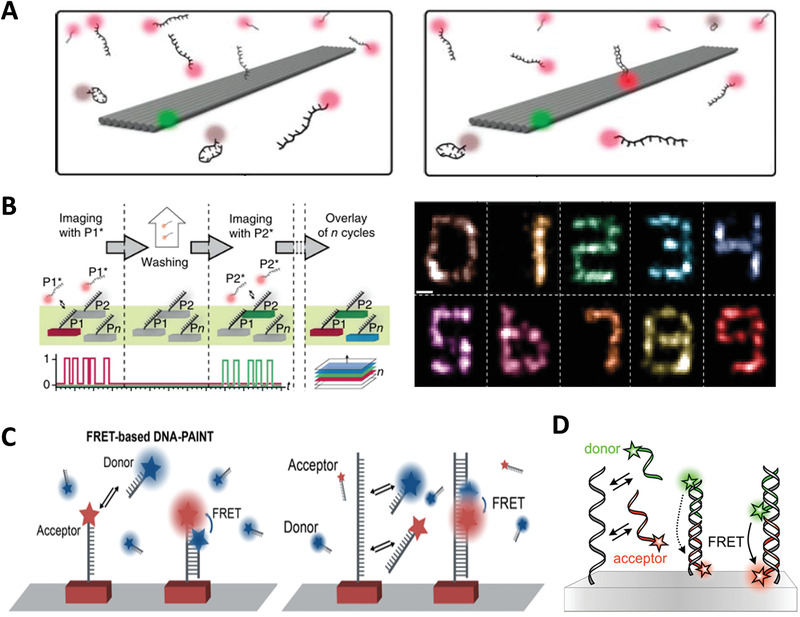
DNA‐PAINT. A) DNA‐PAINT. Reproduced with permission.^[^ ^16^ ^]^ Copyright 2010, American Chemical Society. B) Exchange‐PAINT, and pseudocolor images of ten different origami structures displaying digits 0–9 in one sample. Reproduced with permission.^[^ ^17^ ^]^ Copyright 2014, Nature Publishing Group. C) Initial FRET‐PAINT. Reproduced with permission.^[^ ^207^ ^]^ Copyright 2017, American Chemical Society. D) FRET‐PAINT that relies on the distance dependency of FRET. Reproduced with permission.^[^ ^208^ ^]^ Copyright 2018, American Chemical Society.

DNA‐PAINT was proposed by Jungmann and co‐workers first (**Figure** **11**A).^[^ ^16^ ^]^ DNA‐PAINT is a super‐resolution microscopy technique that utilizes stochastic blinking caused by transient binding of short dye‐labeled oligonucleotides (imaging strands) and protruding docking strands fixed on the target. DNA‐PAINT has higher resolution (<30 nm), extraordinary imaging efficiency (≈95%) and the ability for monitoring dynamic processes and kinetics of DNA moleculars. In DNA‐PAINT technology, DNA origami nanostructures are not only the imaging objects but also excellent tools for investigating the efficacy of DNA‐PAINT and simulating imaging.

In 2014, Jungmann's group developed DNA‐PAINT to a multiplexing approach (exchange‐PAINT) that allowed successive imaging of multiple targets by a single dye (Figure 11B).^[^ ^17^ ^]^ The multiple imaging was performed by iterative washing steps in which imaging strands P1* of the previous target were removed and imaging strands P2* were introduced to image the second target. Roll on in cycles till imaging of all targets was finished. Four‐color 2D and three‐color 3D cellular imaging and ten‐color imaging of origami were obtained by exchange‐PAINT. However, it is obvious that multiple imaging is time‐consuming, and the fluorescent background generated by free diffusion of imaging strands in solution interferes with target imaging. To overcome these problems, Jungmann's group combined FRET to DNA‐PAINT called FRET‐PAINT.^[^ ^207^ ^]^ Originally, docking strands were labeled with an acceptor dye, while imaging strands were tagged with a donor dye (Figure 11C). Nevertheless, nonreplenishment of acceptor dye might lead to permanent photobleaching of the acceptor. In order to realize constant replenishment of acceptor dye, the imaging strands were designed to consist of an acceptor strand and a donor strand. Signal was produced when transient binding of acceptor and donor strands transient simultaneously bind to docking strand. The improved FRET‐PAINT was demonstrated with high‐quality super‐resolution imaged below 30 s. According to the specific distance dependency of FRET, FRET‐PAINT enabled multiplexed imaging (Figure 11D).^[^ ^208^ ^]^ It is worth noting that the binding sites would be damaged by reactive oxygen species (ROS) that produced by excited fluorescent dyes during the binding of imaging strands and docking strands.^[^ ^209^ ^]^ By adding oxygen scavenging reagents into the buffer or positioning the fluorescent dye at a location far from the binding site, that problem can be effectively alleviated.

Based on the short time of imaging, ultrahigh resolution, multiple imaging and immunity to photobleaching, DNA‐PAINT and its derivative techniques overcome many technical difficulties of super‐resolution microscopy and have broad application prospects in cellular imaging.^[^ ^210^ ^]^ DNA‐PAINT can combine with other super‐resolution techniques.^[^ ^211^ ^]^ However, using DNA‐PAINT for living cell imaging has challenges because it is not easy to accurately locate the docking strands to the target in living cell. The docking strands can be bound to the target via the ligands, such as antibody,^[^ ^17^ ^]^ small molecule,^[^ ^212^ ^]^ bacterial‐derived protein,^[^ ^213^ ^]^ etc. When the intracellular targets need to be accurately binded or labeled, large sized ligands are nonapplicable. Therefore, the development of small molecules is vital for DNA‐PAINT in super‐resolution intracellular imaging.

## 6 DNA Robots

DNA nanostructures with reasonable geometric shape, precise spatial addressable ability, obvious biocompatibility, and stability are promising candidates for drug delivery. There are generally four methods for DNA nanostructure to load drugs, including noncovalent linkage, covalent linkage, base complementary pairing, and supramolecular interaction. Each of them has unique advantages. Noncovalent linkage is usually achieved by intercalating small payloads into DNA helix utilizing special DNA double helix structure^[^ ^214^ ^]^ or encapsulating payloads in closed DNA nanostructures with a certain cavity.^[^ ^101^ ^]^ Covalent linkage is normally applied in conditions that therapeutic molecules can be directly linked to the oligonucleotide by chemical reaction.^[^ ^215^ ^]^ Some gene therapy molecules such as siRNAs are directly linked in DNA nanostructures using base complementary pairing.^[^ ^216^ ^]^ Supramolecular interaction usually refers to receptor–ligand interaction.^[^ ^217^ ^]^ Here, DNA robots are DNA nanostructures with certain intelligent behavior, which protect drugs from being interfered before getting the site and release drugs autonomously in designated site, thereby greatly reducing toxic side effects. Such intelligent behavior is generally realized by lock–key systems, in which the logic gate is the major design materials.

**Figure 12:**
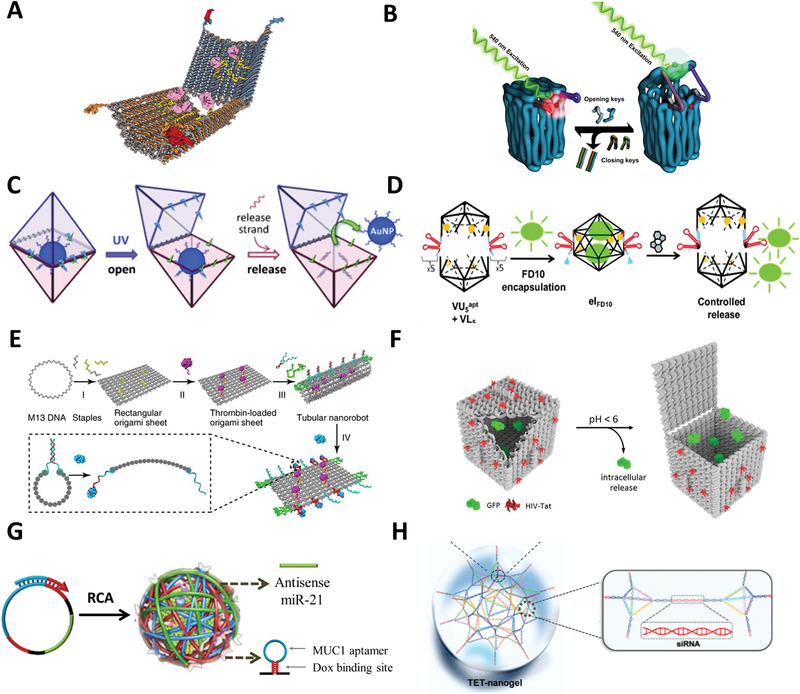
DNA robots for cargo delivery. A) Logic gated DNA nanorobots. Reproduced with permission.^[^ ^22^ ^]^ Copyright 2012, American Association for the Advancement of Science. B) Switchable 3D DNA origami box. Reproduced with permission.^[^ ^219^ ^]^ Copyright 2012, American Chemical Society. C) Light‐responsive DNA nanocapsule with open/close system. Reproduced with permission.^[^ ^85^ ^]^ Copyright 2014, Wiley‐VCH Verlag. D) Controlled release of encapsulated cargo from a DNA icosahedron using a chemical trigger. Reproduced with permission.^[^ ^220^ ^]^ Copyright 2013, Wiley‐VCH Verlag. E) Thrombin‐functionalized DNA nanorobot that exposes payloads when lock strand aptamer binds to target. Reproduced with permission.^[^ ^23^ ^]^ Copyright 2018, Nature Publishing Group. F) DNA origami inside‐out viruses for drug delivery. Reproduced with permission.^[^ ^101^ ^]^ Copyright 2018, American Chemical Society. G) DNA nanosponge for chemo–gene combination therapy. Reproduced with permission.^[^ ^224^ ^]^ Copyright 2019, American Chemical Society. H) A DNA nanogel for siRNA delivery. Reproduced with permission.^[^ ^225^ ^]^ Copyright 2019, The Royal Society of Chemistry.

Logic gate is the basic algorithm of computer. In biological environments, as naturally occurring programmable molecules, DNA is an excellent material for biological logic gate. Logic gate constructed by DNA hereinafter is called DNA logic gate. DNA logic gate mainly relies on the characteristic of nucleic acid molecules, such as strands displacement and cleavage, high affinity of aptamer, and structural transformation of functional strands. AND, NOT, and OR gates are the basic logic gates. DNA logic gate endows DNA robots with smarter behaviors, combining diagnostic sensors for complex signals with controllable release or access to the payload. In 2012, Douglas et al. created a nanorobot in the form of a hexagonal barrel that was controlled by an aptamer‐encoded logic gate (**Figure** **12**A).^[^ ^22^ ^]^ The addressable nanoscale DNA boxes with controllable lids also embodied the concept of logic gate (Figure 12B).^[^ ^13^, ^218^, ^219^ ^]^ DNA logic gate is widely used not only in DDSs, but also in the field of detection and analysis.^[^ ^149^, ^150^ ^]^

In 2014, Takenaka's group prepared a square bipyramidal DNA nanocapsule with a photoresponsive system containing several pairs of Azo‐DNA strands (Figure 12C).^[^ ^85^ ^]^ Cargo was encapsulated into nanocapsule via strand hybridization. When exposed to UV light, Azo‐DNA dissociated, which caused the nanocapsule open for intracellular cargo release via strand displacement. A similar hollow DNA nanocapsule also successfully achieved controllably encapsulating and releasing of cargos autonomously (Figure 12D).^[^ ^220^ ^]^ The DNA icosahedron was formed by combining upper and lower halves via ligand‐sensitive modules. The module was an aptamer folded into a special shape. Once aptamers bound to ligands, the reconfiguration of aptamers caused icosahedron to dissociate into two halves, thereby releasing cargos into cells. Aptamer is a powerful tool in targeted DDSs, and it have a huge advantage in the DNA nanostructure‐based DDSs. Recently, Li et al. constructed an intelligent DNA robot which can find tumor‐associated blood vessels, and then release thrombin to form thrombus to “starve” the tumor (Figure 12E).^[^ ^23^ ^]^ The nanorobot consisted of a flat rectangular DNA origami sheet with thrombin loaded through strands hybridization. Then, the sheet was rolled into a hollow tube by fastener strands to prevent thrombin from exposing before reaching the tumor vessel. The fastener strands containing antinucleolin aptamer sequences AS1411 acted as both targeting domains and molecular triggers. The combination of AS1411 and nucleolin caused the fastener strands to disassociate, thus exposing thrombin. A series of animal experiments demonstrated that the nanorobot not only inhibited the tumor growth, but also prevented the formation of tumor metastasis. This DNA robot is a fully autonomous DNA robotic system for accurate drug delivery and targeted therapy, achieving big advance in cancer therapy aided by DNA origami technique.

Anionic DNA exhibits poor penetration into negatively charged cell membrane due to strong electrostatic repulsion. To solve this problem, DNA nanostructures were usually conjugated with proteins targeting cellular receptors,^[^ ^221^ ^]^ complexed with hydrophobic drugs,^[^ ^222^ ^]^ or modified with virus capsid proteins,^[^ ^223^ ^]^ etc. Burns et al. introduced an everted‐virus DNA topology to deliver functional protein (Figure 12F).^[^ ^101^ ^]^ As is shown in Figure 11F, the cube with a pH‐responsive lid was noncovalently decorated with HIV‐Tat transduction domains that conferred effective infectivity on its exterior, and functional proteins were encapsulated into it. The DNA cube differs from the boxes mentioned above,^[^ ^13^, ^218^ ^]^ because it dose not need externally keys. The hollow cube directly opened in acidic endogenous environments, thereby facilitating the release of proteins. Compared to chemotherapeutic drugs, genomic drug molecules can serve as building materials for constructing DNA assemblies due to their homology. Zhang et al. realized chemo–gene combination therapy through the DNA nanosponge that formed by co‐assembly of the long ssDNA sequences (containing MUC1 aptamers, antisense miR‐21s, as well as sequences for Dox loading) and magnesium pyrophosphate during the RCA reaction (Figure 12G).^[^ ^224^ ^]^ The collapse of nanosponge induced by the dissolution of magnesium pyrophosphate in acid lysosome exposed antisense miR‐21s and released Dox. In another study, therapeutic siRNA was used as a linker in a TDN‐based DNA nanogel (Figure 12H).^[^ ^225^ ^]^

The ultimate goal of targeted DDS is to deliver the drug to a designated site of the body, thus minimizing side effects on normal tissues. In the absence of protection, the method of intercalating drugs into DNA helix is difficult to prevent the leakage of drugs before reaching the destination, thereby causing damage to normal cells. Considering stability or targeting, DNA nanorobots with autonomous behavior are ideal carriers for targeted delivery. However, there are a few issues that need to be noticed. The structure of DNA origami played an important role in drug loading efficiency. For example, flexible DNA origami structure susceptible to structural fluctuations exhibited lower loading efficiency.^[^ ^226^ ^]^ DNA is a endogenous substance, which is theoretically nontoxic and harmless, but the amount of DNA in cells is always fixed. So the consequences of a large amount of extra DNA entering the cells must be figured out. Although DNA robots as drug carriers have been validated in animal experiments, there is still a long way to go for DNA robots being used in clinical trials.

## 7 Conclusions and Future Outlooks

In the past two decades, the development of new DNA machines for biomedical applications has become a hot topic in nanoscience and nanotechnology. The unique properties and multiple functionalities of DDAs have been validated in numerous researches. In this review, we summarized the designing, mechanisms, and biomedical applications of DDAs. The assembly methods and computer‐aided software for DDAs designing are listed, and the advantages and disadvantages of each software are discussed. DDAs can perform structural transformations or predictable behaviors in response to corresponding stimuli. The mechanisms of dynamic behaviors of DDAs are classified into exogenous stimuli, such as ions, light, temperature, pH, DNA strands, electric and magnetic field, and endogenous stimuli, such as glutathione, nucleic acids, pH, proteins, and adenosine triphosphate. The special dynamic behaviors of DDAs, such as DNA walkers, DNA hydrogels, DNA nanochannels, DNA‐PAINT, and DNA‐robots in biomedical applications, are summarized. Precise single molecule customization and strong biocompatibility make DDAs a powerful tool to accurately organize and manipulate molecules. However, there are several challenges need to be addressed at the moment.

First, the in vivo stability of DNA is poor. DNA is highly susceptible to hydrolysis, oxidation, and degradation by nucleases. High salt concentrations and ambient temperatures are usually required to maintain structural integrity of DNA nanostructures, which severely limits its applicability in biologically relevant environments because of the poor stability under physiological salt conditions. It has been reported that DNA nanostructures covering with protein (bovine serum albumin and class II hydrophobin),^[^ ^227^ ^]^ peptide,^[^ ^228^ ^]^ and lipid membranes^[^ ^37^ ^]^ exhibited better stability in vivo. Additionally, it is demonstrated that the ability of Na^+^, Mg^2+^, sperminem, and oligolysine of length ten (K_10_) in stabilizing DNA origami is enhanced in turn. Appropriate modification protects the DNA structure so it can improve its stability. But the key to stability enhancement is to make the structure more compact. For example, it was recently declared that UV can induce the formation of C—C covalent bond between T or C bases, and the stability of the irradiated DNA object was improved a lot both in high temperature and low salt concentration.^[^ ^229^ ^]^ Apart from poor stability in vivo, the possibility of strand breaking introduced by long length of M13mp18, the hindrance caused by hairpin in M13mp18 in folding, and the expensive synthesis cost of substantial staple strands also need to be considered.

Second, the cellular internalization ability of DNA is low. Cellular uptake efficiency, drug load/release content, toxicity, and metabolism are criterias for assessing the delivery capacity of DNA nanostructure‐based DDSs. However, strong electrostatic repulsion between both negatively charged DNA nanostructures and cell membranes lead to low internalization rate. Wang et al. found that larger structures showed higher cellular uptake efficiencies, which might be resulted from enhanced surface interactions.^[^ ^230^ ^]^ Bastings et al. concluded that there is a significant linear relationship between compactness of DNA nanostructures and cell internalization efficiency. Solid DNA nanostructure was better absorbed by cells than the hollow and wireframe structures.^[^ ^231^ ^]^ Additionally, it is reported that DNA origami structures were preferentially accumulated in the kidneys of normal mice and mice with rhabdomyolysis‐induced acute kidneys injury (AKI).^[^ ^232^ ^]^ All in all, these studies revealed that the internalization efficiency, transportation, and metabolism of DNA nanostructures were highly related with the geometry, size, density, modification, aspect ratio of the structure, as well as cell lines. Therefore, to enhance the internalization, charge eversion, modification of targeted molecules, improvement of structural density, etc., should be considered.

Third, endosomal escape of DDAs is a problem. The intracellular routes of DNA molecules differs with different DNA size, cell types, etc. According to previous studies, DNA assemblied are internalized by cells via a caveolae‐mediated endocytosis, and finally transported to lysosome‐like cellular compartments through early and late endosomes.^[^ ^230^, ^233^ ^]^ Liang et al. declared that the small DNA tetrahedron was transported in a microtubule‐dependent manner.^[^ ^233^ ^]^ However, in most cases, the statement of caveolin‐dependent pathway was only verified in certain cells. Although many researches were based on this pathway so far, the mechanism of internalization and transportation of DNA nanostructure still remains to be comprehensively investigated. Additionally, it was observed that drugs in DNA nanostructures were hard to escape from endosomes or lysosomes into the cytosol,^[^ ^230^ ^]^ which is a big biological challenge in DDSs. Biomimetic materials have an advantage in endosomal escape, such as erythrocyte membrane, cancer cell membrane, virus, etc. Proton‐responsive cationic liposome was used to realize enhanced lysosomal escape of siRNA.^[^ ^234^ ^]^ A DNA‐MoS_2_ nanosheets superstructure with ATP responsiveness also exhibited good lysosomal escape of drugs.^[^ ^235^ ^]^ Accelerating the release of drugs or changing the endocytosis of DDAs are effective ways to enhance lysosomal escape. Therefore, the combination of DNA nanostructures and biomimetic materials is worth studying.

Four, kinetics of DDAs remains to research. It is unquestionable that the character of DNA nanostructure is critical for its application, especially for DDAs. For example, the elastic property of ssDNA is vital to the movement of DNA walker, the torsional buckling of dsDNA is essential to the construction of DNA hydrogels, and the tensile and bending properties and pressure‐proof capacities of DNA nanostructure are significant to its stability. The force yielded by ssDNA and dsDNA under tension depends on their length, base sequence periodicity, and geometric structure. For instance, the persistence length of DNA double crossover is approximately twice of normal DNA helix.^[^ ^5^ ^]^ Complex applications of DDAs such as DNA probe require an deep understanding of the mechanical response, not just basic elastic properties. There are various competitive relationships, like key–lock system, toehold‐mediated strand displacement, the ligand and complementary strands competitively bond to aptamer, etc. Only by accurately calculating the force response, can the response unit be triggered. In addition, there is motion, especially long‐term motion, the flexibility, and softness of flexible components determine the range and time of motion that DDAs can withstand. Virtual model that can be used to prescreen hypothetical origami is prior to experimental implementation to assist the design process, judging the feasibility of putative structure, thereby reducing deviation. Engel et al. explored the dynamics of force propagation in the structure by forcibly unraveling DNA origami through a virtual model.^[^ ^236^ ^]^ Besides, detailed characterization of the fundamental structural property of DNA nanostructure can also be obtained via simulation.^[^ ^237^ ^]^ Therefore, it is necessary to study the mechanical property of DNA structure. Further, with the development of RNA technology, RNA nanostructures have also been developed. RNA enables self‐fold during transcription, thereby can be encoded and expressed in cells. A single‐stranded RNA nanostructure has been demonstrated to be constructed both by annealing folding in vitro and cotranscriptional folding.^[^ ^238^ ^]^ An RNA octahedron was also successfully assembled by origami technique.^[^ ^239^ ^]^ RNA as a building material is promising because the function of RNA is extensive, and the amount of cellular RNA can fluctuate in a wide range, thus RNA structures also have advantages in DDSs.

All in all, DDAs have shown great potential in the field of diagnosis, therapy, imaging, biosensing, construction of intelligent machine, etc. Functional nucleic acid molecules endow DNA nanostructures with multiple behaviors. Moreover, various chemical modifications on DNA allow the generation of composite nanostructures. Strategies will be developed to overcome the challenges and accelerate the application of DNA assemblies, making DDAs as promising tools for biomedical applications.

## Conflict of Interest

The authors declare no conflict of interest.
